# Efficient Discovery of Potent AKR1B10 Inhibitors Using Co-folding Model Boltz‑2

**DOI:** 10.1021/acs.jcim.6c00874

**Published:** 2026-09-12

**Authors:** Shinya Kawano, Akira Ikari, Satoshi Endo, Kentaro Tomii

**Affiliations:** † Laboratory of Biochemistry, 12784 Gifu Pharmaceutical University, Gifu, Gifu 501-1196, Japan; ‡ Artificial Intelligence Research Center (AIRC), National Institute of Advanced Industrial Science and Technology (AIST), Koto-ku, Tokyo 135-0064, Japan; § United Graduate School of Drug Discovery and Medical Information Sciences, Gifu University, Gifu, Gifu 501-1194, Japan; ∥ Center for One Medicine Innovative Translational Research (COMIT), Gifu University, Gifu, Gifu 501-1193, Japan; ⊥ Preemptive Food Research Center, Gifu University, Gifu, Gifu 501-1193, Japan

## Abstract

Although affinity prediction of protein–ligand binding remains an important challenge, cofolding models are expected to make virtual screening more effective for drug discovery and development. To verify the effectiveness of selecting a tractable number of candidates from a compound library using cofolding models, we strove to identify a novel inhibitory active compound using Boltz-2, a representative cofolding model, for aldo-keto reductase 1B10 (AKR1B10), which is highly expressed in various cancers. Consequently, of the 40 candidate compounds obtained after narrowing-down 867 candidates from a chemically diverse library based on prediction results by Boltz-2 and candidate selection with sufficient diversity, 70% (28 of 40 tested) compounds at IC_50_ < 10 μM were found to have inhibitory activity and to provide identification of multiple submicromolar inhibitors exhibiting novel scaffolds. Our results demonstrate that our sparse selection approach using Boltz-2 is helpful for enhancing AI-driven drug discovery. Moreover, the findings highlight its potential applicability for translating AI-generated predictions into experimentally actionable hits.

## Graphical abstract

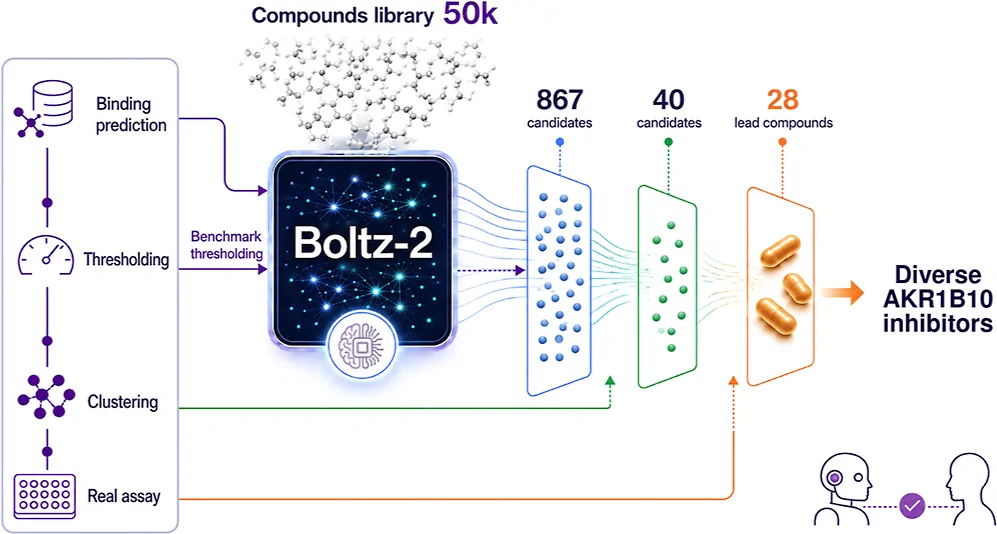

## 1 Introduction

The field of in silico virtual screening has undergone a major paradigm shift during the past decade, transitioning from classical physics-based docking approaches to deep learning-driven structure prediction and generation frameworks.^1,2^ Conventionally used docking tools such as AutoDock Vina and Glide have long been centrally important for structure-based drug design. However, these methods often rely on simplified scoring functions and static receptor representations, consequently limiting their ability to capture induced-fit effects, long–range interactions, and the complex energetic landscape governing protein–ligand recognition.^3,4^

Recent advances in “co-folding” models, as exemplified by AlphaFold 3, RoseTTAFold All-Atom (RFAA), and the Boltz-1/2 series, have expanded the scope of computational drug discovery considerably by enabling the joint modeling of protein–ligand­(−cofactor) complexes directly from sequence and SMILES representations.^5–9^ These models employ invariant point attention and related architectures to generate three-dimensional atomic coordinates while accounting for backbone and side-chain flexibility.^6,10,11^ In principle, such frameworks offer a unified solution to structure prediction and ligand pose generation. Among them, Boltz-2 can reportedly predict protein–ligand binding affinity approximately 1000 times faster than standard free-energy perturbation (FEP) calculations, while maintaining comparable accuracy to that provided by FEP, based on large amounts of data for the binding affinity.^6^

Despite their impressive predictive capabilities, a crucially important limitation of those models has been reported: The good predictability and accuracy of the cofolding models are limited to cases that are similar to the trained data set, i.e., known protein–ligand interactions. Based on results of recent rigorous benchmarks, a salient concern is that those models might depend heavily on memorization of training data for protein–ligand interactions.^12^ This requirement can hamper effective applications for de novo drug design and can pose a major bottleneck in translating AI-generated predictions into experimentally actionable hits. However, according to a recent report, Boltz-2 demonstrated superior ability to distinguish true positives from false ones.^13^

For this study, we set a biologically meaningful task as an example of the prediction target, Aldo-keto reductase 1B10 (AKR1B10), to confirm the effectiveness of Boltz-2 for prediction of binding affinity, especially for novel compounds. AKR1B10 is a cytosolic enzyme with a conserved (α/β)_8_-barrel fold. It is involved in the detoxification of reactive carbonyl species and lipid metabolism. It has emerged as a clinically relevant biomarker and therapeutic target in multiple malignancies, including hepatocellular carcinoma and non-small cell lung cancer.^14–17^ Moreover, AKR1B10 has been demonstrated to play a crucially important role in cancer cell proliferation, migration, progression, and the development of anticancer drug resistance.^17–21^ Consequently, this enzyme has attracted considerable attention as a promising therapeutic target for cancer treatment.

During recent decades, our laboratory has established a comprehensive chemical library of AKR1B10 inhibitors, identifying a wide spectrum of potent and selective agents. These include natural products such as unsaturated fatty acids,^22^ mangostins,^23^ terpenoids,^24^ curcuminoids,^25^ and steroids,^26^ as well as synthetic scaffolds including chromene derivatives^17,27,28^ and caffeic acid derivatives.^29^ It is noteworthy that AKR1B10 inhibitors derived from natural products are not regarded as drug-like and that challenges persist in their clinical application. Novel anticancer drugs are also expected to increase the sensitivity of cancer cells to existing drugs and to overcome resistance to them.

Therefore, we attempted to identify an inhibitory active and ‘novel’ compound with a slightly modified approach of standard virtual screening using Boltz-2 for AKR1B10 as a proof of concept. For this study, we verified the effectiveness of selecting candidate compounds from the library using Boltz-2, with the aim of using it in accurate and detailed assays for a small number of compounds (a few dozen), mainly in the early to midstage of drug discovery. Specifically, of the 40 candidate compounds obtained by narrowing down candidates from a chemically diverse library of 50,240 compounds with thresholds for predictive values determined based on results of benchmarks for AKR1B10 and candidate selection with sufficient diversity, 70% (28 out of 40 tested compounds, derived from the 867 Boltz-2 candidates through score-based and diversity-based filtering) at IC_50_ < 10 μM and 17.5% (7 out of 40) at IC_50_ < 1 μM were shown to have inhibitory activity and to identify multiple inhibitors exhibiting novel scaffolds. Interaction between these 40 candidate compounds and the target protein was also monitored using thermal shift assay. This orthogonal assay further validates the prediction results provided by Boltz-2 and allows us to confirm that the selected compounds contain not only simple binders but also potent inhibitors for AKR1B10. Our results demonstrate that our sparse selection approach based on identified compounds with Boltz-2 can enhance hit enrichment considerably, mitigate the so-called "dry–wet" gap, and transform all-atom generative models into robust tools for complex enzymatic targets.

## 2 Results

### 2.1 Retrospective Benchmarking Using Boltz-2

To evaluate the ability of Boltz-2 to discriminate AKR1B10 inhibitors from nonbinders, we first performed a retrospective benchmark analysis. A data set was assembled consisting of 132 known inhibitors curated from ChEMBL and 6600 decoy molecules generated using the DUD-E protocol.^30,31^ Among 132 inhibitors, only 43 compounds with pChEMBL values ≥7.0 against AKR1B10 (corresponding to ≤100 nM potency for IC_50_/*K*_i_) were regarded as actives, whereas compounds with pChEMBL values below this threshold were treated as negatives in the retrospective benchmark (Figure 1A). When multiple activity annotations were available for a given compound, the most potent reported value (maximum pChEMBL) was used for label assignments.

**Figure 1:**
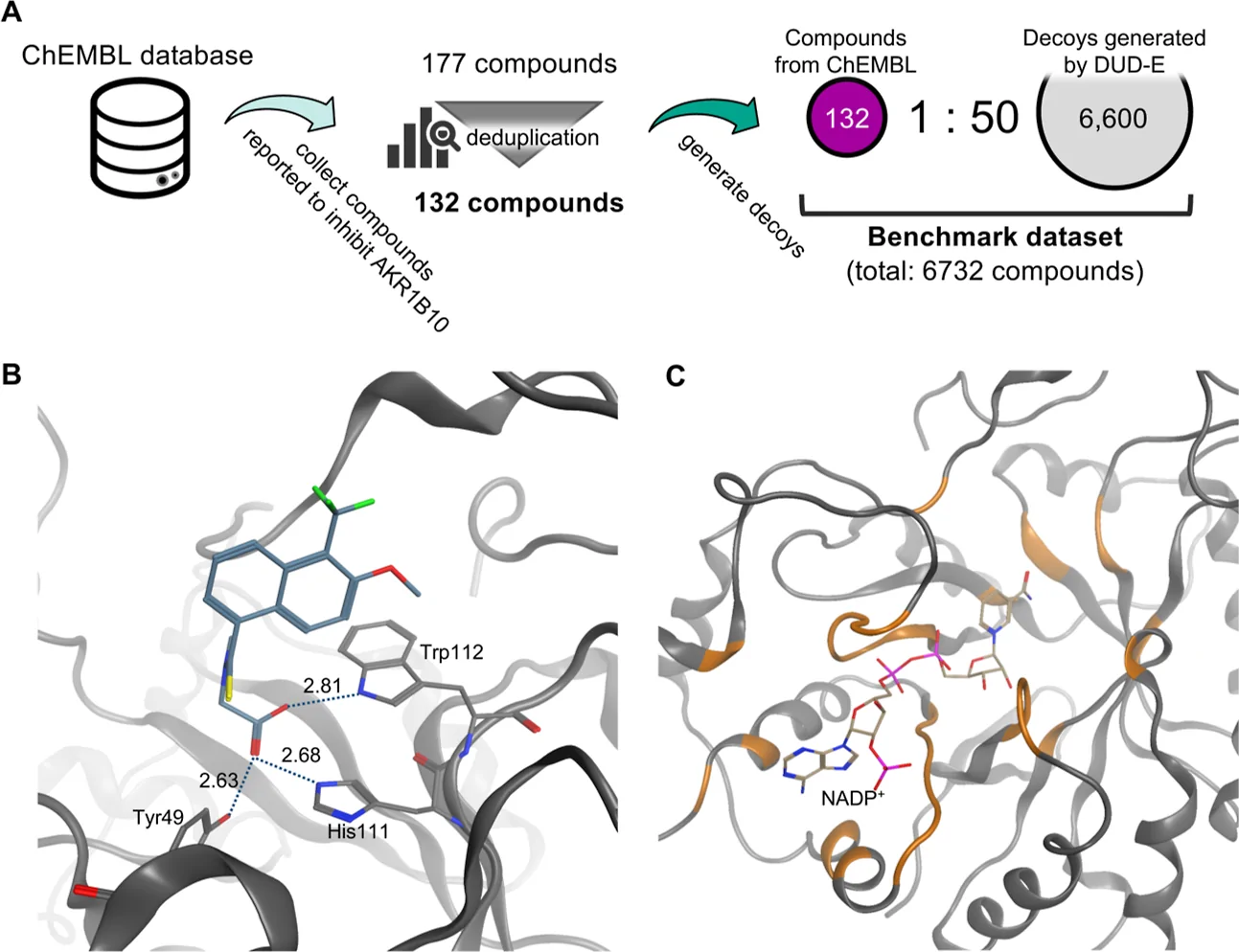
Preparation of the benchmark data set and definition of structural constraints for AKR1B10. (A) Schematic workflow for the construction of the retrospective benchmark data set, comprising 132 known inhibitors from the ChEMBL database and 6600 decoys generated using DUD-E (1:50 ratio), totaling 6732 compounds. (B) Identification of key active-site residues using the crystal structure of the AKR1B10–NADP^+^–tolrestat complex (PDB ID: 1ZUA). Distances (in Å) emphasize the catalytic triad: Tyr49, His111, and Trp112. (C) Definition of the cofactor binding pocket (residues in orange) based on amino acids within 4.5 Å of the cocrystallized NADP^+^.

Distance-based constraints for targeting the catalytic residues Tyr49 and His111, along with the adjacent Trp112 which dictates pocket specificity, were derived from our extensive discovery data and were introduced to the screening calculations by Boltz-2 (Figure 1B). In addition, residues within 4.5 Å of the cocrystallized NADP^+^ were used to define the cofactor-binding pocket (Figure 1C). For internal validation of the constrained inference process, tolrestat (IC_50_ = 54 ± 4 nM) was evaluated as a reference compound. Across replicate Boltz-2 runs, tolrestat consistently yielded extremely high scores, confirming both the robustness of the model and the validity of the imposed catalytic constraints (Figure 2A).^28,32^

**Figure 2:**
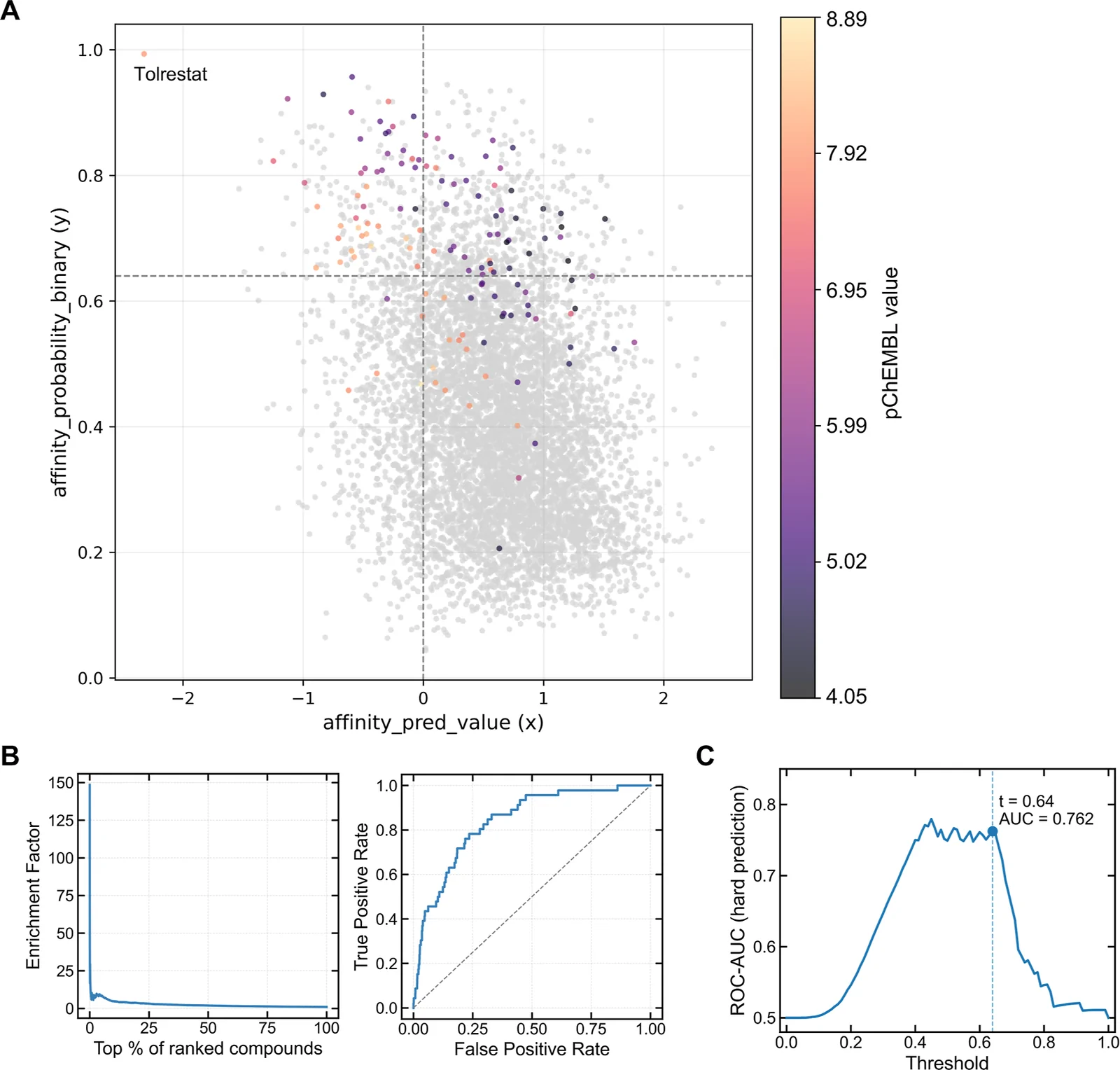
Retrospective benchmarking and data-driven threshold determination. (A) Known inhibitors, colored by pChEMBL value, and DUD-E decoys, shown in gray, are plotted in the affinity_pred_value (X) and affinity_probability_binary (Y) plane; the reference compound tolrestat is indicated. (B) Enrichment factor (EF) and ROC-AUC curve for the benchmark data set. (C) ROC-AUC for hard predictions as a function of the binary threshold *t*. The selection threshold was set at *t* = 0.64, marked by the dashed line, corresponding to the local maximum AUC of 0.762.

Under these conditions, Boltz-2 demonstrated robust classification performance, achieving an overall ROC-AUC value of 0.84 (Figure 2B and Table 1) in terms of binary classification of active compounds vs inactive ones. It is noteworthy that all 132 known inhibitors obtained from ChEMBL used as the active set can be fully included in the Boltz-2 affinity-head training data (ChEMBL_34, 2024–03 release; 132/132 compounds appear in ChEMBL with document_year ≤2023; Table S1). Accordingly, the retrospective ROC-AUC of 0.84 and EF_1%_ of 8.7 should be regarded as a sanity check, not as evidence of generalization to unseen actives. This performance is comparable to, or exceeds, that of conventional physics-based docking approaches, which often struggle with AKR family enzymes because of the pronounced flexibility of the specificity pocket.^33,34^ Consistent with the ROC analysis, Enrichment Factor evaluation showed effective prioritization of active compounds in the top-ranked fraction (EF_1%_ = 8.7).^35^

**Table 1:**
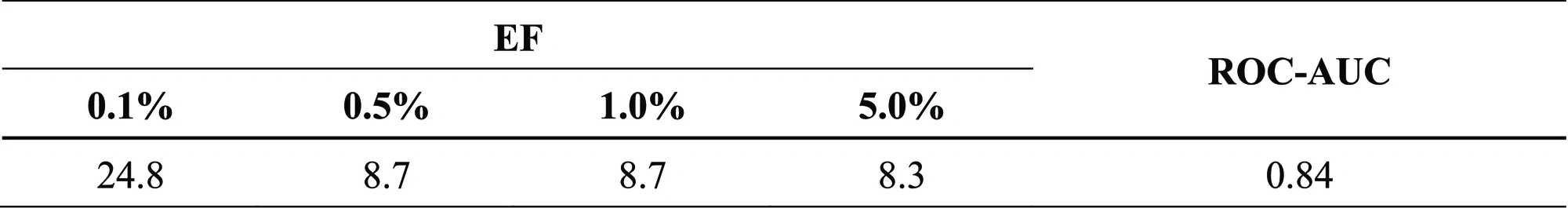
Retrospective Benchmark Performance Metrics**.** Enrichment Factors (EF) Calculated for the Top 0.1%, 0.5%, 1.0%, and 5.0% of the Ranked Library, Alongside the Overall ROC-AUC Value

To define a stringent and nonarbitrary criterion for prospective screening, the affinity_probability_binary threshold *t* was scanned systematically from 1.00 to 0.00. Examination of the ROC-derived performance profile revealed a local optimum at *t* = 0.64 (Figure 2C). Such ROC-guided threshold selection strategies have been demonstrated as effective for balancing false-positive suppression and hit recovery in highly imbalanced virtual screening settings.^36–38^ Therefore, we confirmed that the enrichment of true actives is expected when using affinity_probability_binary = 0.64 and affinity_pred_value = 0 as thresholds. Because the same data set was used both to tune *t* and to calculate the retrospective metrics, these values should be viewed as threshold-optimized estimates without held-out validation; an independent test of *t* = 0.64 is its prospective application to the Enamine Discovery Diversity Set described below.

### 2.2 Prospective Screening and Chemical Space Analysis

The validated protocol was subsequently applied to the Enamine Discovery Diversity Set comprising 50,240 "lead-like" compounds. Of these, 867 compounds (approximately 1.7% of the set) that exceeded the predefined thresholds were identified as primary candidates (Figures 3A and S1) by enforcing constraints targeting the catalytic machinery using Boltz-2. From this subset, 51 compounds were selected using hierarchical clustering based on the Tanimoto distance to maximize chemical space coverage while minimizing experimental redundancy (Figure 3B).^39,40^ Of these, 40 compounds (approximately 0.08% of the set; Table 2) were subjected to the following assays, according to their availability. This marked reduction supported a highly focused and experimentally tractable validation strategy.

**Figure 3:**
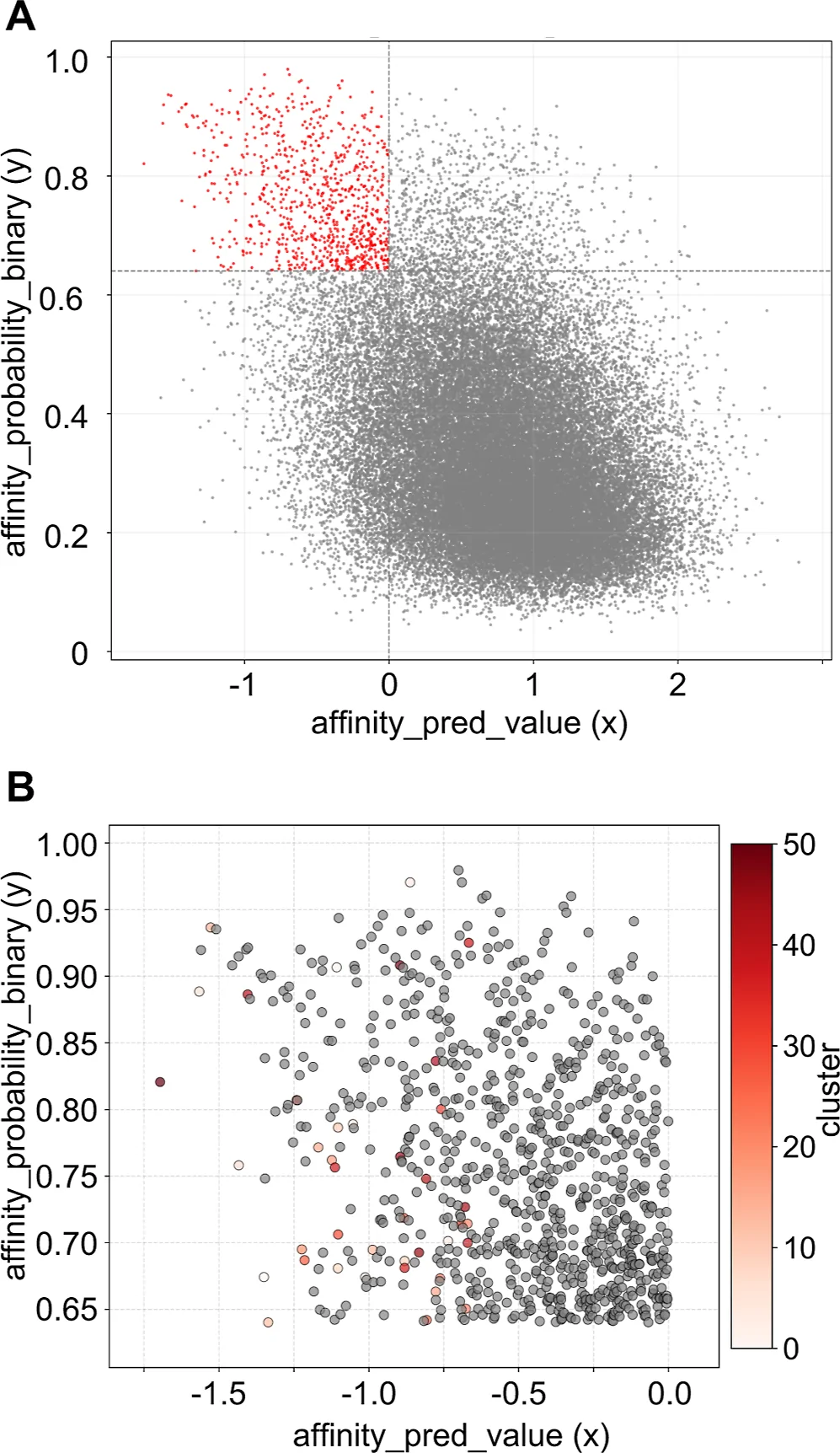
Prospective virtual screening and diversity-based selection. (A) Global distribution of Boltz-2 prediction scores for the Enamine Discovery Diversity Set (50,240 compounds). Red dots represent the 867 primary hits satisfying affinity_probability_binary ≥0.64 and affinity_pred_value ≤0. (B) Selection of 40 representative compounds for validation by experimentation. Circles are colored by cluster ID based on hierarchical agglomerative clustering of Morgan2 fingerprints, ensuring maximum coverage of the chemical space.

**Table 2:** Computational Prediction Scores for the Validated AICE Serie**s** ^a^

compound	affinity_pred_value	affinity_probability_binary
AICE001	–1.6962435	0.82056755
AICE002	–1.5656369	0.88840073
AICE003	–1.527707	0.93650389
AICE004	–1.4336677	0.75806904
AICE005	–1.4039853	0.88629329
AICE006	–1.3498023	0.67418277
AICE007	–1.3354908	0.64022201
AICE008	–1.2406188	0.8069495
AICE009	–1.2232172	0.69498372
AICE010	–1.214054	0.68690145
AICE011	–1.1683089	0.77147126
AICE012	–1.1230527	0.76197749
AICE013	–1.1128044	0.75645495
AICE014	–1.1068842	0.90658796
AICE015	–1.1028786	0.68076599
AICE016	–1.1025312	0.78637815
AICE017	–1.1022414	0.70618874
AICE018	–1.0526483	0.7889123
AICE019	–1.0117054	0.67403483
AICE020	–0.9872012	0.69471359
AICE021	–0.8957697	0.90818745
AICE022	–0.8951669	0.76450586
AICE023	–0.8826039	0.71854729
AICE024	–0.880402	0.6863097
AICE025	–0.8803775	0.68111002
AICE026	–0.8619592	0.97037494
AICE027	–0.8322724	0.6926049
AICE028	–0.8086249	0.74795973
AICE029	–0.8066767	0.64194274
AICE030	–0.7765346	0.66328216
AICE031	–0.7763465	0.83632904
AICE032	–0.7616206	0.67329574
AICE033	–0.7586004	0.80017722
AICE034	–0.7353655	0.70126706
AICE035	–0.691956	0.71384454
AICE036	–0.6785107	0.72679281
AICE037	–0.6758488	0.65043736
AICE038	–0.6702557	0.71422327
AICE039	–0.6698827	0.69985831
AICE040	–0.6661267	0.92504346

a List of affinity_pred_value and affinity_probability_binary for all 40 (AICE001–AICE040) compounds subjected to validation by experimentation.

It is noteworthy that the selected compounds exhibited low mutual similarity based on Morgan2/Tanimoto fingerprints, thereby indicating marked topological diversity, while displaying moderate similarity in terms of extended reduced graph (ErG) fingerprints (Figure S2). ErG fingerprints emphasize pharmacophoric feature arrangements rather than scaffold identity. Therefore, this pattern suggests that Boltz-2 did not converge on candidates based on simple 2D structural proximity. Instead, the model preferentially identified chemically diverse scaffolds that satisfied the spatial and functional interaction patterns required by the AKR1B10 active site, as described in 2.4 hereinafter.

### 2.3 Experimental Validation

Enzymatic assay and thermal shift assay (TSA) were conducted to ascertain which of the 40 identified compounds were true hits (active compounds). These analyses reveal that the predictions produced by Boltz-2 extend beyond mere binding compounds, encompassing compounds that exhibit inhibitory activity.

Enzymatic assays for the 40 compounds revealed an exceptionally high hit rate of 70% (28 out of 40 compounds) at a screening concentration of 10 μM, as defined by an inhibition rate of at least 50% (Figure 4A). It is noteworthy that this 70% value represents the hit rate within the 40 compounds derived from the 867 primary Boltz-2 candidates through score-based and diversity-based filtering, not an unbiased estimate of the full 867-compound pool. This rate markedly exceeds the 1–10% hit rates commonly reported for traditional structure-based virtual screening campaigns.^41^ Even at 1 μM, 7 of the 40 compounds (17.5%) retained measurable inhibitory activity. Several submicromolar inhibitors were identified, including AICE003 (AICE stands for AKR1B10 Inhibitor Candidate Enrichment), with an IC_50_ value of 197 ± 12 nM (Figures 4B,C, S3, and Table 3).

**Figure 4:**
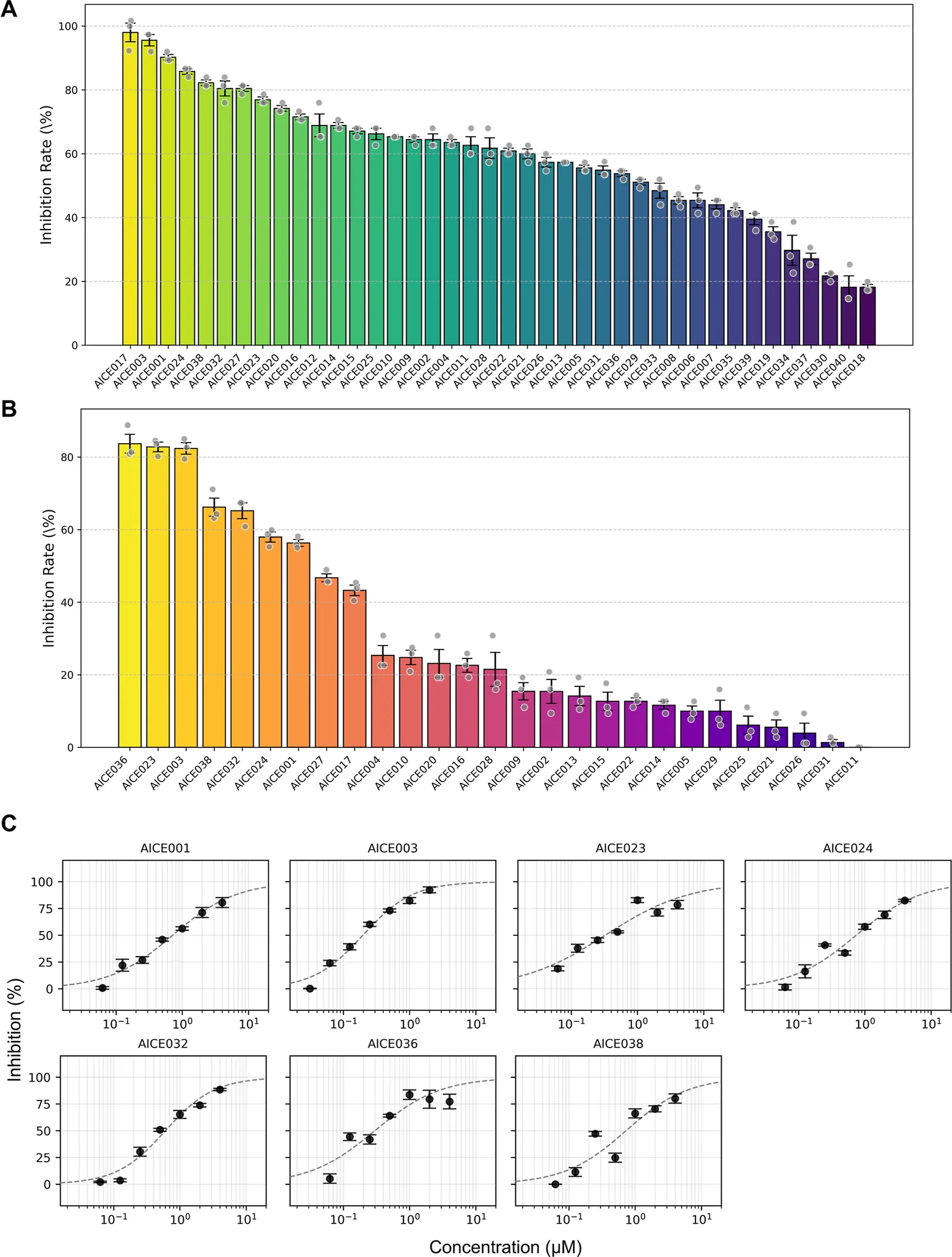
Experimental evaluation of AKR1B10 inhibitory activity. (A) Primary screening results for the 40 selected AICE compounds at 10 μM. A hit rate of 70% (28/40) was achieved based on *a* ≥ 50% inhibition threshold. (B) Secondary screening at 1 μM for compounds that exhibited high potency in the primary assay. (C) Representative dose–response curves for the most potent inhibitors. Inhibition data were fitted to a four-parameter logistic model. Data points represent the mean ± SEM from at least three independent experiments (*n* ≥ 3).

**Table 3:** Potency of Representative AKR1B10 Inhibitor**s** ^a^

compound	IC_50_ AKR1B10 (μM)	**IC**_**50**_ **AKR1B1 (μM)**	selectivity ratio (B1/B10)	predicted Trp112 conf	selectivity class
AICE001	0.717 ± 0.050	3.95 ± 0.28	5.51	B10	B10-selective
AICE003	0.197 ± 0.012	0.54 ± 0.04	2.75	B10	B10-selective
AICE017	1.283 ± 0.087	1.02 ± 0.17	0.80	B1	non-selective
AICE023	0.325 ± 0.040	75.24 ± 19.44	231.2	B10	B10-selective
AICE024	0.743 ± 0.073	4.04 ± 0.95	5.44	B10	B10-selective
AICE032	0.604 ± 0.042	0.84 ± 0.16	1.39	B10	non-selective
AICE036	0.298 ± 0.045	0.82 ± 0.15	2.75	B10	B10-selective
AICE038	0.728 ± 0.111	0.35 ± 0.05	0.48	B1	B1-selective

a IC_50_ values for human AKR1B10 (seven sub-micromolar AICE inhibitors plus AICE017, which served as the IC_50_-confirmed B1-conformation reference) and for human AKR1B1, expressed as mean ± SEM (μM) from independent triplicates (NADPH consumption assay). The AKR1B1/AKR1B10 selectivity ratio and the predicted Trp112 side-chain conformation in the top-ranked Boltz-2 model of the AKR1B10–ligand complex ("B10" = un-flipped ground state; "B1" = AKR1B1-like flipped state) are presented in the right-hand columns. Selectivity class: B10-selective (ratio ≥2), non-selective (0.5 < ratio <2), B1-selective (ratio ≤0.5).

The complexity of interpreting binding events for deep-learning-derived hits was highlighted further by the TSA results. Although several potent inhibitors such as AICE003, AICE001, and AICE032 induced clear protein stabilization, others exhibited decreased thermal stability (e.g., AICE023 and AICE024) or negligible Δ*T*_m_ shifts (e.g., AICE036 and AICE038) (Figure S4). These discrepancies observed between the TSA profiles and enzymatic inhibition potencies are consistent with the findings of Kabir et al. on aldo-keto reductase family members.^42^ That study established that, although thermal shift assays (Δ*T*_m_) can track the thermodynamic stabilization of AKR1B10, they do not necessarily yield a strict, quantitative linear correlation with enzymatic IC_50_ values. This nonlinear relation stems from the distinct chemical scaffolds of the hits, which can induce diverse conformational stabilization profiles or affect the binding environment of the NADP^+^ cofactor differentially.^43,44^ The primary strategic objective of incorporating TSA in this workflow was to obtain direct, orthogonal evidence verifying that the predicted ligands physically bind to the target protein, thereby confirming genuine target engagement and ruling out assay artifacts. Therefore, TSA was positioned as a key qualitative filter to substantiate physical interactions, whereas the definitive determination of functional inhibitory efficacy was driven rigorously by steady-state enzymatic IC_50_ evaluation.

### 2.4 Structural Novelty of Hit Compounds and Predicted Binding Modes

Similarity heatmap analyses (Figure 5) demonstrated that the AICE compound series is topologically distinct from earlier reported inhibitors registered in ChEMBL. This observation was supported further by Tversky similarity analysis (Figures S5 and S6).^45,46^ The low Morgan2-based Tanimoto coefficient values (*T* < 0.35) show that Boltz-2 achieved scaffold hopping successfully.^38,46^ At the same time, moderate similarity in ErG fingerprint space suggests preservation of pharmacophoric arrangements that are important for target engagement. By integrating both topology-based and feature-based similarity metrics, we confirm that the protocol can identify structurally novel scaffolds and exclude compounds that would otherwise occupy the binding pocket in catalytically inert orientations.^32,47^

**Figure 5:**
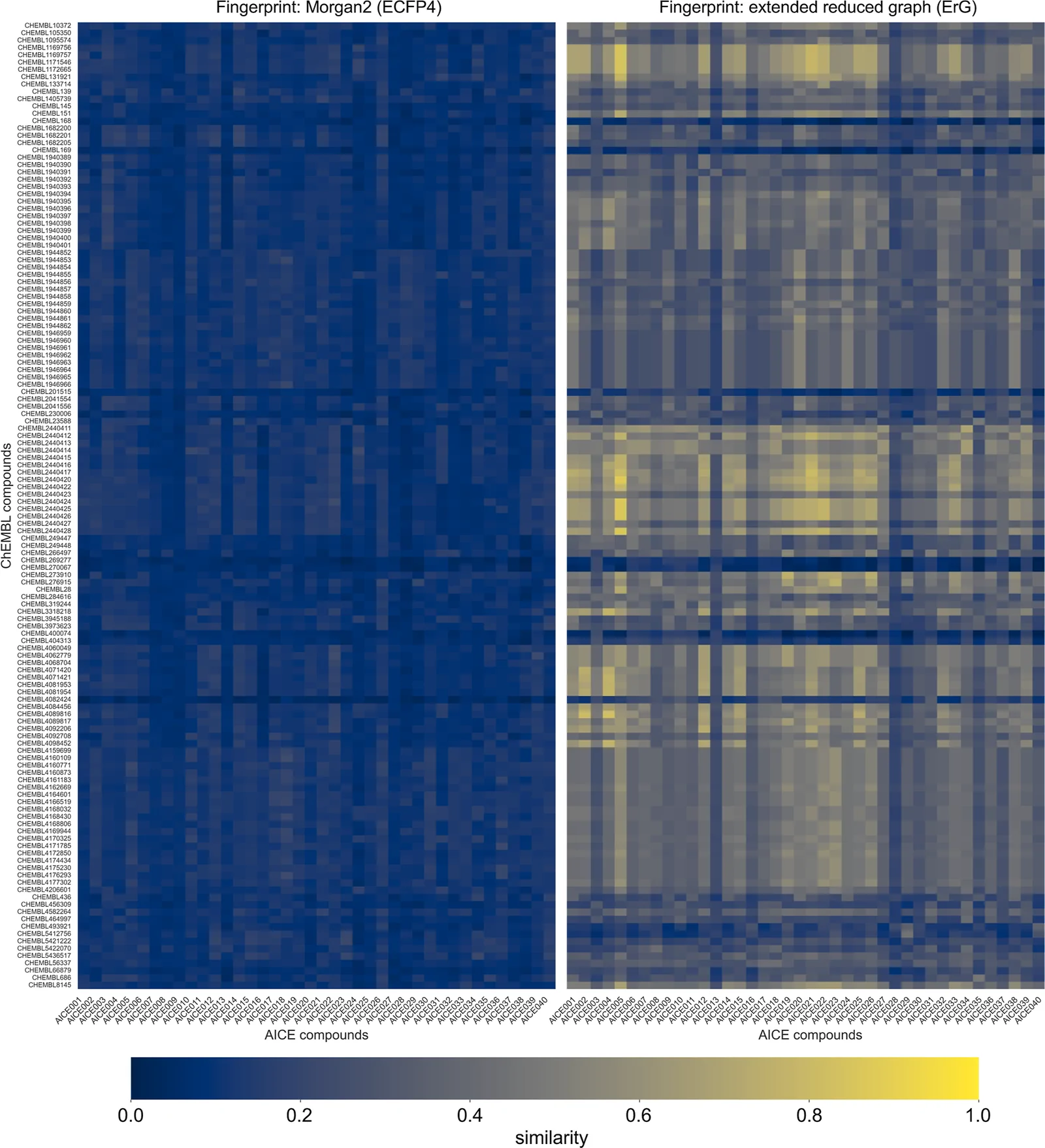
Structural novelty and pharmacophoric diversity analysis. Similarity heatmaps show the Tanimoto coefficient (*T*) between 40 AICE hits (*x*-axis) and 132 known AKR1B10 inhibitors from the ChEMBL database (*y*-axis). Fingerprints used for the comparison are Morgan2 (ECFP4; left) and extended reduced graph (ErG; right). The color scale represents similarity values of 0.0 to 1.0.

Inspection of predicted binding modes for representative compounds AICE003, AICE023, and AICE036 (Figure 6A–C) confirmed preservation of hydrogen bonds with anionic site residues within the AKR1B10 active site. In each case, polar functional groups were positioned at distances that were compatible with hydride transfer relative to the nicotinamide ring of NADP^+^. The groups were stabilized by interactions with Tyr49 and His111. Consistent with the native structural features of the enzyme, the top-ranked Boltz-2 prediction models for AICE003, AICE023, and AICE036 showed that all these ligands leave the Trp112 side chain in its original, unflipped conformation, designated as the “B10-position”. In these models, the “B10-position” is highly stabilized by a specific Gln114-centered hydrogen-bond network (Figure 6D). By contrast, the reference crystal structure of the AKR1B10–tolrestat complex (PDB ID: 1ZUA) shows that the bulky tolrestat induces an atypical side-chain flip of Trp112 into the “B1-position”. This flipped state mimics the expanded pocket topology of the closely related AKR1B1 isoform and often compromises isoform selectivity. As the AICE series binds while preserving the native “B10-position” without triggering this cross-reactive induced-fit adaptation, it is thought that these potent compounds preferentially exploit the ground-state architecture unique to AKR1B10. This allows them to function as isoform-specific inhibitors.^32,47^

**Figure 6:**
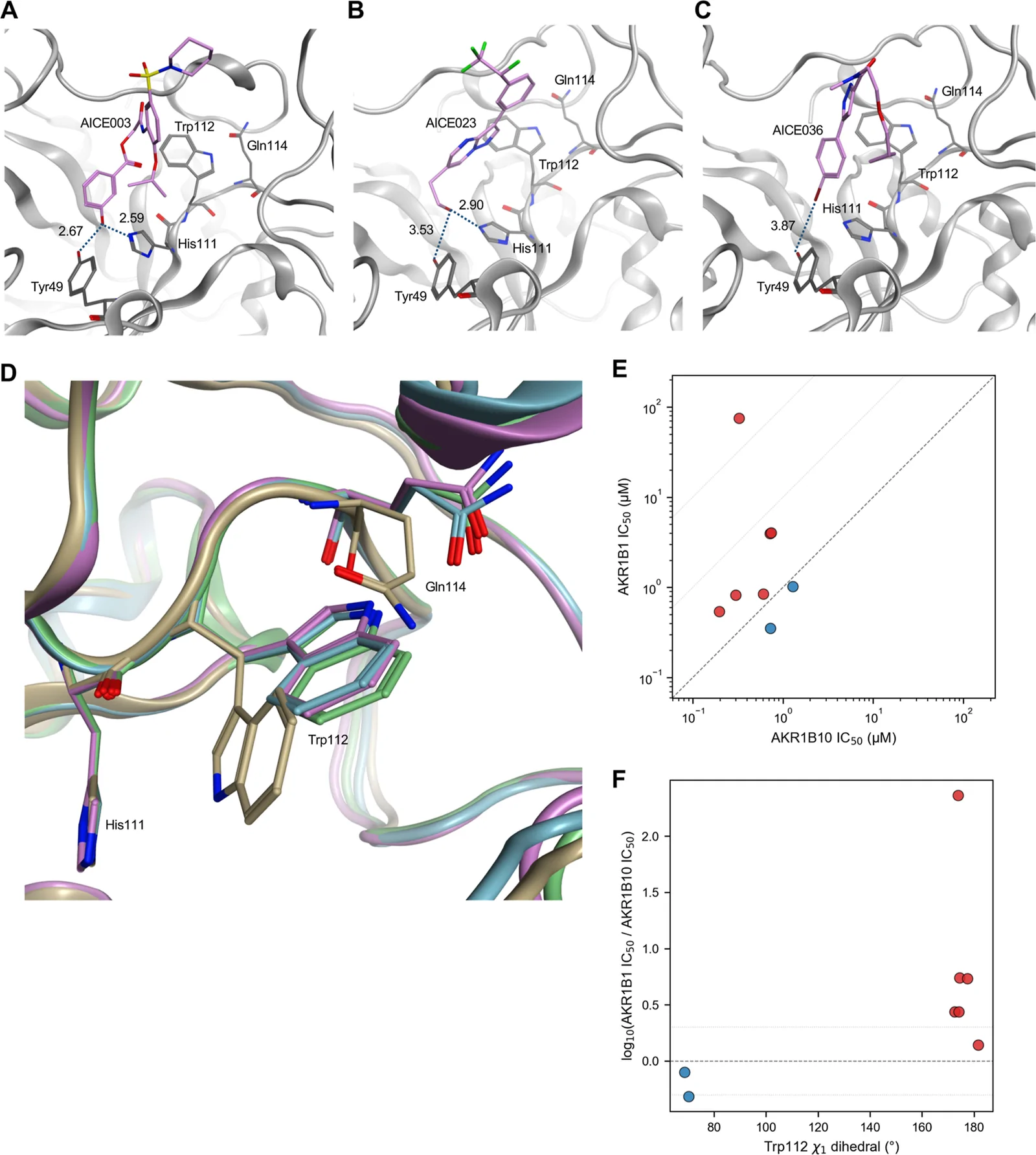
Predicted binding modes of representative hit compounds. Molecular models of (A) **AICE003**, (B) **AICE023,** and (C) **AICE036** (shown as sticks) in complex with AKR1B10 (represented as gray cartoons) as predicted using Boltz-2. Key hydrogen-bonding interactions with the catalytic residues (Tyr49 and His111) are shown by dashed lines. (D) Superposed and close-up view of Trp112 in the three predicted models and 1ZUA. The top ranked models with AICE003 (cyan), AICE023 (magenta), and AICE036 (light sea green) and the crystal structure of the AKR1B10–NADP^+^–tolrestat complex (1ZUA; gold) are shown. (E) Boxplot of the AKR1B1/AKR1B10 selectivity ratio for the eight IC_50_-confirmed AICE compounds (AICE001, 003, 017, 023, 024, 032, 036, 038), grouped by the Boltz-2-predicted Trp112 side-chain conformation: six B10-conformation compounds (unflipped ground state) and two B1-conformation compounds (AKR1B1-like flipped state). Geometric-mean selectivity is 6.47-fold (range 1.39–231) for the B10 group and 0.62-fold (range 0.48–0.80) for the B1 group, corresponding to a 10.4-fold systematic shift between the two groups. (F) Scatter plot of IC_50_ against AKR1B1 (*y*-axis) and IC_50_ against AKR1B10 (*x*-axis) (μM, log–log scale) for the same eight compounds. The diagonal denotes equipotency; B10-conformation compounds (filled circles) lie above the diagonal (AKR1B10-favored), whereas B1-conformation compounds (open circles) lie below the diagonal. IC_50_ values are the mean ± SEM (*n* ≥ 3) determined using a spectrophotometric NADPH consumption assay.

To test this structural hypothesis by experimentation, AKR1B1 inhibition assays were performed on the eight AICE compounds (AICE001, 003, 017, 023, 024, 032, 036 and 038) with confirmed IC_50_ values under conditions matched to the AKR1B10 assay. The measured AKR1B1/AKR1B10 selectivity ratios were divided clearly according to the predicted Trp112 conformation by Boltz-2: The six B10-conformation compounds yielded a geometric mean selectivity ratio of 6.47 (range 1.39–231) in favor of AKR1B10, whereas the two B1-conformation compounds yielded a geometric mean ratio of 0.62 (range 0.48–0.80) in favor of AKR1B1. The two conformational groups therefore differ by a systematic 10.4-fold shift in selectivity (Figure 6E,F and Table 3). AICE023 (B10-position) exhibited 231-fold AKR1B10 selectivity, whereas AICE038 (B1-position) showed inverse selectivity at 0.48-fold. This selectivity provides direct experiment-derived support for the previously described structural rationale. The single nonselective B10 example (AICE032, 1.39-fold) suggests the presence of a secondary determinant beyond Trp112, possibly loop-C tip residues (Val301 and Gly302). This particular point is expected to be the subject of follow-up work. It is noteworthy that the Trp112 χ^1^ torsion angle, which distinguishes the two conformations, is not restrained by the Boltz-2 pocket contact. Therefore, the B10/B1 split is a ligand-dependent free prediction by the model. Moreover, its alignment with the measured selectivity is a nontrivial validation. These results support the predicted binding modes of AICE compounds.

### 2.5 Robustness of the Prospective Hit Rate

To ascertain whether the 70% prospective hit rate reflects Boltz-2 enrichment rather than an effect of diversity selection or library curation, we performed three complementary analyses. First, stratification of the 40 tested compounds by their cluster size (Table S2) as small (≤5), medium (6-15), and large (≥16) respectively yielded hit rates of 81.2%, 60.0% and 66.7%, all above 50%, with fundamentally no correlation between cluster size (logarithmic scale) and percentage inhibition at 10 μM (*r* = −0.020, *n* = 40; Figure S7). Second, a cluster-independent nearest-neighbor density metric (Tanimoto *T* = 0.3–0.7) showed |*r*| < 0.30 with percentage inhibition at 10 μM across all five thresholds (Figure S8), indicating that the effect is not specific to the Morgan2/Tanimoto/k = 51 clustering scheme. Third, to separate Boltz-2-specific enrichment from any effect of library curation, we extended the 51-cluster solution to 251 clusters across the full 50,240-compound library. After exclusion of the 40 AICE compounds, the threshold-pass rate (binary probability ≥0.64 and predicted log10 IC_50_ < 0) was 8.80% (784/8911) in the 40 AICE-occupied clusters, compared with 0.10% (43/41,076) in the 211 AICE-free clusters, corresponding to an 88-fold enrichment (one-sided Mann–Whitney *U* test, *p* = 6.4 × 10^–42^; Figure 7A). The AICE-free clusters showed a pass rate 17-fold below the library-wide value (1.65%, excluding the 40 AICE compounds), whereas the AICE-occupied clusters (8951 compounds; 17.8% of the library) included 94.8% of all threshold-passing compounds. The distribution of AICE-occupied regions across chemical space is shown in Figure 7B. Consistent with this, hit-anchored hierarchical clustering of the seven sub-μM AICE inhibitors and their nearest library neighbors placed the anchors on independent branches, with a median Tanimoto coefficient of approximately 0.10 to the full library (Figures S9 and S10).

**Figure 7:**
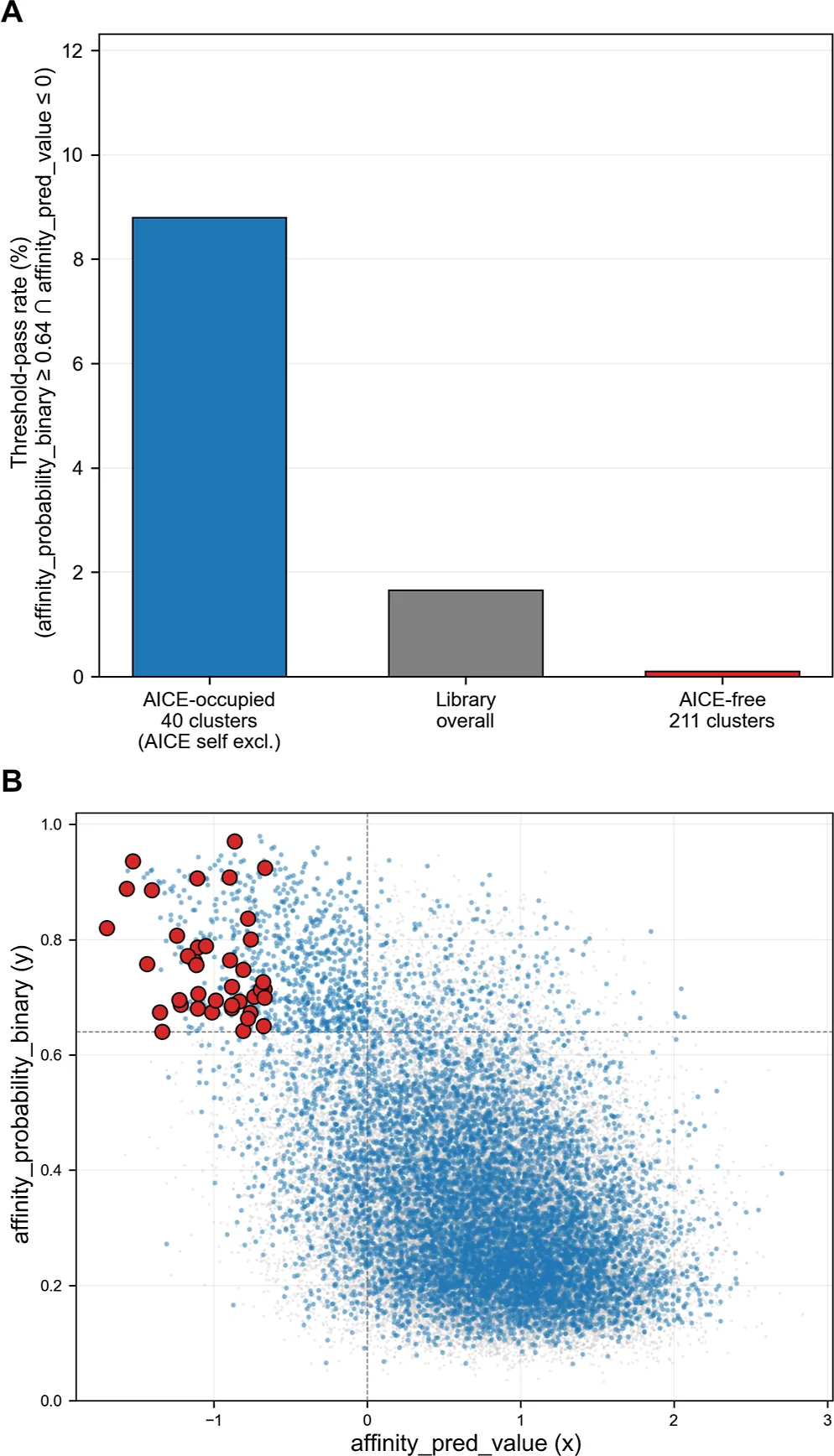
Robustness of the prospective hit rate against cluster-based selection and library curation. (A) Threshold-pass rate (affinity_probability_binary ≥0.64 and affinity_pred_value ≤0) in the 40 AICE-occupied clusters versus the 211 AICE-free clusters of the 50,240-compound Enamine Discovery Diversity Set after seeded extension of the 51-cluster solution to 251 clusters; the 40 AICE compounds themselves were excluded before scoring. The pass rates are 8.80% (784/8911) for the AICE-occupied clusters and 0.10% (43/41,076) for the AICE-free clusters, corresponding to an 88-fold enrichment (Mann–Whitney *U* one-sided *p* = 6.4 × 10^–42^). (B) Two-dimensional projection of the full 50,240-compound library showing the distribution of AICE-occupied chemical regions (8951 compounds, 17.8% of the library; colored), which account for 94.8% of all threshold-passing compounds, against AICE-free regions (gray). The figure illustrates that AICE-occupied clusters are not concentrated in a single subregion of chemical space but are spread across the diversity space.

### 2.6 Correlation of Boltz-2 Scores and Confidence Metrics with Experimental Activity

Correlation analysis between experimentally measured inhibition rates and Boltz-2 prediction scores revealed only weak linear relations, with Pearson’s *r* values of −0.35 to 0.08 (Figure 8). This analysis was extended to eight Boltz-2 confidence metrics (confidence_score, ptm, iptm, ligand_iptm, complex_plddt, complex_iplddt, complex_pde, and complex_ipde) for the 40 AICE compounds and the 132 ChEMBL retrospective compounds, correlated with TSA Δ*T*_m_, percentage inhibition at 10 μM and 1 μM, pIC_50_, and p*K*_*i*_. No strong positive correlation was found. ptm and complex_plddt showed moderate negative correlation with TSA Δ*T*_m_ (ptm *r* = −0.50, complex_plddt ρ = −0.52; Figure S11), whereas correlation with IC_50_ and p*K*_*i*_ was consistently weak (|*r*| < 0.30 throughout; Figure S12 for p*K*_*i*_ of the 132 known inhibitors).

**Figure 8:**
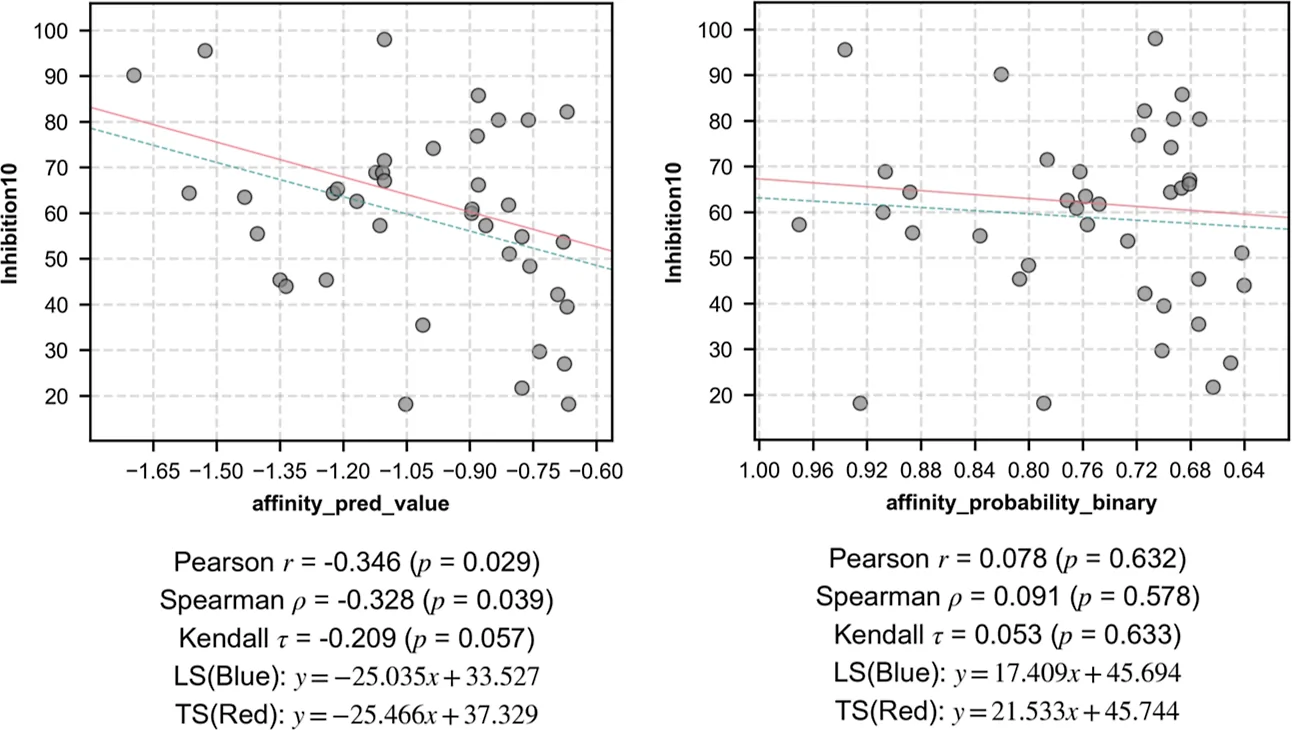
Correlation analysis between computational scores and experimentally measured activity. Scatter plots present the respective relations between inhibition rates at 10 μM and affinity_pred_value (left) and affinity_probability_binary (right). Pearson correlation coefficients indicate weak linear correlation, which is consistent with the model’s performance as a robust classifier rather than a precise ranking tool.

## 3 Discussion

### 3.1 True Hit Rate

Of the 40 tractable candidate compounds obtained by narrowing-down with sufficient diversity from the top hits using Boltz-2, 70% (28 compounds) exhibited inhibitory activity at IC_50_ < 10 μM and 17.5% (7 compounds) at IC_50_ < 1 μM. Is the success rate attributable to overlapping with the training data? Regarding the viewpoint of ligands, at least 40 compounds possess novel scaffolds against known AKR1B10 inhibitors, according to similarity analysis of them. At the same time, moderate similarity in ErG fingerprint space indicates that the pharmacophoric arrangements are preserved to some extent. This preservation suggests that known inhibitor–protein interaction patterns can yield useful information related to the spatial arrangements and constraints for scaffold hopping using Boltz-2.

The robustness analyses indicate that the reported hit rate reflects Boltz-2 enrichment within a chemically coherent subspace, rather than reflecting other factors. The hit rate was independent of the cluster size. The conclusion was unchanged across clustering methods. Moreover, the 88-fold enrichment of threshold-passing compounds in AICE-occupied versus AICE-free clusters showed that the lead-/drug-like curation of the Enamine library alone is insufficient to explain the observed performance, although the diversity selection might improve chemical-space coverage.

### 3.2 Pose Prediction

In the aldo-keto reductase (AKR) superfamily, productive catalysis requires precise positioning of the substrate relative to the nicotinamide ring of the NADP^+^ cofactor to enable hydride transfer within a narrow distance and angular window.^32,48^ Empirical insights gained from our historical inhibitor discovery campaigns served as the foundation for the construction of our constraint-guided predictive framework, ensuring that the AI-driven search space remained aligned with the functional requirements of AKR1B10. Specifically, AKR1B10 uses catalytically important residues, Tyr49 and His111,^32,49^ along with the adjacent Trp112 dictating pocket specificity,^47^ which are often overlooked by generalized generative models.^50^ By enforcing proximity to Tyr49, His111, and Trp112 explicitly, the present approach might exclude compounds that would otherwise occupy the binding pocket in catalytically inert orientations. Therefore, those three residues are used for setting constraints in this study. This approach restricts ligand placement to the intended binding site in most cases.

Despite extensive efforts, achieving high isoform selectivity against the closely related AKR1B1, which shares approximately 71% sequence identity, remains challenging.^17,47^ However, as described above, the protocol enriched compounds possessing essential binding features while maintaining scaffold diversity, thereby suppressing nonproductive binding modes that typically contribute to the dry–wet gap.^51^ Even minor deviations in ligand orientation can abolish catalytic competence, rendering many predicted binding modes functionally inert. As such, AKR enzymes represent a stringent benchmark for evaluating the functional fidelity of generative structure prediction models. Although the present study specifically examines AKR1B10, the constraint-guided framework is readily extensible to other enzyme targets for which catalytic geometry and electrostatic compatibility are critical determinants of productive binding.

### 3.3 Limitations of Score-Based Ranking

Weak correlation between the Boltz-2 scores/confidence metrics and the experimental activity described above is partly attributable to the compressed dynamic range of the tested data set because all evaluated compounds were drawn from the high-score regime (*t* ≥ 0.64). Under such input-variable variance compression, Pearson *r* and Spearman ρ tend to be attenuated relative to the true population values. Accordingly, the correlation observed for the AICE 40 set should be interpreted as lower-bound estimates of the predictive value of the confidence metrics rather than direct measurements of it. A more rigorous evaluation that includes both true binders and true nonbinders remains as future work. In addition, single-point inhibition measurements at 10 μM tend to approach saturation, thereby masking more subtle differences in binding affinity. This behavior exemplifies the well-documented “scoring function paradox”, whereby computational models classify binders versus nonbinders effectively but fail to rank relative affinities accurately among active compounds.^52–54^ Such limitations are often attributed to incomplete treatment of solvation effects and entropy–enthalpy compensation in tight-binding regimes.^52,55,56^

In fact, the primary strength of our approach lies in hit enrichment rather than fine-grained affinity ranking. As described above, quantitative affinity predictions remain challenging to the degree that our approach to maximize chemical space coverage while minimizing redundancy among top hits detected by Boltz-2 or other cofolding models presents an appropriate and practical way to find novel bioactive chemotypes in drug discovery.

Several further limitations should be discussed.

(i) Data leakage: as noted above, the 132 known inhibitors used in the retrospective benchmark can be included in the Boltz-2 affinity training set. Therefore, the retrospective ROC-AUC of 0.84 reflects not only structure-based discrimination but also memorization. It should be interpreted as a sanity check, whereas evidence for generalization comes from the prospective screen and the postcutoff Sun 2024 benchmark (Figure S13),^57^ which are not included among the training data for Boltz-2.
(ii) Threshold tuning: A threshold of *t* = 0.64 was selected as a local maximum in the ROC-AUC scan of 132 known AKR1B10 inhibitors obtained from ChEMBL and 6600 decoy molecules and subsequently evaluated on the same data set; accordingly, the retrospective performance metrics represent threshold-optimized values without held-out validation. Because this tuning procedure is based on target-specific actives rather than on library composition, the same threshold might be expected to generalize to alternative synthetic libraries with similar physicochemical properties. However, when the same *t* = 0.64 was applied to the postcutoff, leakage-free Sun 2024 actives, only 1 of 5 compounds exceeded the threshold (median binary_prob, 0.43; maximum, 0.68; Table S3). These results, which indicate reduced recall for chemotypes not represented among the 132 known inhibitors, suggest that application of this framework to a new target would require an analogous target-specific tuning step.
(iii) Library curation: The prospective evaluation used the precurated Enamine Discovery Diversity Set. Although the 88-fold enrichment in AICE-occupied versus AICE-free clusters suggests that the high hit rate is not a uniform property of the curated library, generalizability to noncurated synthetic libraries with a broader physicochemical distribution remains to be tested.
(iv) Clustering dependence: The Morgan2/Tanimoto/*k* = 51 procedure was kept fixed throughout. The cluster-independent nearest-neighbor density cross-check confirms that the main conclusions are independent of this specific clustering choice.

## 4 Conclusion

For this study using a tractable number 40 of compounds selected from the library by narrowing down the plausible hits by Boltz-2, we achieved an experimentally validated hit rate of 70% within the diversity-filtered and score-filtered representative set, greatly exceeding those typically reported for conventional virtual screening approaches. The successful identification of submicromolar inhibitors, including AICE003, with topologically novel scaffolds highlights the capacity of this approach to achieve scaffold hopping while preserving catalytic compatibility. Furthermore, AKR1B1 inhibition assays of the eight AICE compounds showed a 10.4-fold difference in selectivity between the predicted B10 and B1 conformation groups, providing experiment-derived support for the Trp112-based selectivity rationale.

Although the present model is more effective at classifying active versus inactive compounds than at accurately ranking binding affinities, its ability to narrow the dry–wet gap considerably represents a meaningful advance for practical AI-driven drug discovery. More broadly, this work underscores the importance of integrating earlier biochemical and mechanistic knowledge into generative AI workflows. Our approach constitutes a rational and extensible strategy for aligning high-capacity deep learning models with the functional requirements of enzymatic targets. Future efforts will specifically examine refinement of the scoring metrics and applying this approach to other cofactor-dependent enzyme systems where catalytic geometry is a dominant determinant of ligand efficacy.

## 5 Materials and Methods

### 5.1 Reference Structure and Constraint Definition

The crystal structure of human AKR1B10 in complex with NADP^+^ (PDB ID: 1ZUA) was used as a reference structure. To guide the generative model toward biologically relevant conformations, two distinct categories of distance-based constraints based on this structure were introduced during Boltz-2 inference.

First, cofactor constraints were defined by identifying all amino acid residues located within 4.5 Å of the cocrystallized NADP^+^ molecule (CCD code: NAP), thereby delineating the cofactor-binding pocket. Second, ligand constraints were imposed by designating key residues involved in catalytic hydride transfer (Tyr49, His111, and Trp112) as ligand-binding anchors. These constraints were designed to restrict ligand placement to orientations that are compatible with the AKR1B10 catalytic environment.

### 5.2 Boltz-2 Prediction Protocol

Virtual screening was conducted using Boltz-2 (ver. 2.2.0; https://github.com/jwohlwend/boltz/commit SHA: ac1c8582d61a728bee899451ff95e6e93be8922a).^6^ For each candidate compound, a YAML input file (Figure S14) was generated containing the AKR1B10 amino acid sequence, the ligand SMILES representation, and the NADP^+^ cofactor. Multiple sequence alignments were generated using MMseqs2 (Release 17-b804f) against the UniRef90 database (ver. 2025_03).^58^ All Boltz-2 inference runs were executed on the ABCI supercomputing infrastructure using default settings, with the potential-based energy term deliberately disabled (use_potentials = off) to keep virtual screening of approximately 50,000 compounds computationally tractable. The physical feasibility of the resulting poses (Table S4), assessed by steric-clash and intraligand overlap checks, and a comparison of use_potentials on versus off (Figures S15 and S16), are provided as Supporting Information.

### 5.3 Performance Metrics and Threshold Determination

To define an objective threshold used for prospective screening, a retrospective benchmark was performed using a curated data set of 132 known AKR1B10 inhibitors obtained from ChEMBL and 6600 decoy compounds generated using the Directory of Useful Decoys (DUD-E) protocol.^30^ Screening performance was quantified using the Enrichment Factor at *x* % (*EF*_*x*_), defined as shown below.

1 $${EF}_{x}=\frac{{Hits}_{x}/N_{x}}{{Hits}_{total}/N_{total}}$$

Therein, Hits_*x*_ and *N*_*x*_ respectively denote the number of hits (active compounds, here) and the number of compounds, identified within the top *x* % of the ranked list. Hits_total_ and *N*_total_ respectively denote the number of active compounds and the number of compounds in the library.^59^ Receiver operating characteristic (ROC) analysis was conducted. The affinity_probability_binary threshold was scanned across its full range from 0.00 to 1.00 in 0.01 increments. The final selection threshold was set as *t* = 0.64, corresponding to the first local maximum of the ROC-area under the curve (ROC-AUC) profile.^35,60^ Separately, compounds were considered predicted actives when their affinity_pred_value was ≤0, a cutoff calibrated to correspond to IC_50_ ≤ 1 μM.

### 5.4 Clustering and Diversity Selection

Primary hits (*n* = 867) obtained from the Enamine Discovery Diversity Set (50,240 compounds) were subjected to diversity analysis. Molecular fingerprints were generated using Morgan fingerprints with radius 2 (Morgan2) and bit length of 2,048, as implemented in RDKit. Hierarchical agglomerative clustering was performed based on the Tanimoto distance (*D*), which is derived from the Tanimoto coefficient (*T*). The Tanimoto coefficient, representing the similarity between bit vectors *A* and *B*, is defined as shown in the following equation.

2 $$T\left(A,B\right)=\frac{\left|A\cap B\right|}{\left|A\right|+\left|B\right|-\left|A\cap B\right|}$$

Therein, |*A*∩*B*| denotes the number of bits set to 1 in both vectors, and |*A*| and |*B*| respectively represent the number of bits set to 1 in vectors *A* and *B*. The Tanimoto distance (*D*) was then calculated as

3 D=1−T

The optimal number of clusters (*k* = 51) was found using the Silhouette score *s*(*i*).

4 $$s\left(i\right)=\frac{b\left(i\right)-a\left(i\right)}{\max\left\{a\left(i\right),b\left(i\right)\right\}}$$

In that equation, *a*(*i*) represents the average intracluster distance for compound *i*, and *b*(*i*) denotes the minimum average distance to compounds in the nearest neighboring cluster.^45,46,61^ Representative compounds were selected as the member with the most favorable Boltz-2–predicted affinity (affinity_pred_value) from each cluster. These representatives were used to maximize the chemical space coverage while minimizing redundancy.

### 5.5 Structural Novelty Analysis

To assess the structural novelty of the identified hits relative to known AKR1B10 inhibitors, the Tversky similarity index (*S*) was calculated using weighting parameters α = 0.8 and β = 0.2, emphasizing substructural differences.^62^

5 $$S\left(Α,Β\right)=\frac{\left|A\cap B\right|}{\left|A\cap B\right|+\alpha \left|A\backslash B\right|+\beta \left|B\backslash A\right|}$$

For that equation, |*A*\*B*| is defined as {*x*∈*A*/*x*∉*B*} and |*B*\*A*| is {*x*∈*B*/*x*∉*A*}.

For each hit *i*, novelty was defined as the complement of the maximum similarity score relative to the ChEMBL reference inhibitor set.

6 $${novelty}_{i}=1-\max_{j\in ChEMBL}S\left(i,j\right)$$

### 5.6 Materials

All 40 test compounds (Table S5) were purchased from Enamine Ltd. with stated purity of at least 96%. The purity and identity of the eight AICE compounds were confirmed by LC–MS analysis provided by the supplier. They were dissolved in methanol. The compound library used for the virtual screening was “Enamine Discovery Diversity Set” (ver. 31 October 2023), which contains 50,240 "lead-like" compounds without PAINS. Recombinant human AKR1B10 protein was prepared as described earlier.^17^ NADPH, NADP^+^, and pyridine-3-aldehyde (P3A) were obtained from commercial suppliers. They were used without further purification.

### 5.7 Enzymatic Inhibition Assay

AKR1B10/AKR1B1 inhibitory activity was evaluated by monitoring NADPH consumption at 340 nm using a spectrophotometric assay.^17^ Experiments were conducted in 96-well flat-bottom plates using a Multiskan SkyHigh microplate spectrophotometer (Thermo Fisher Scientific Inc.). The reaction mixture consisted of AKR1B10/AKR1B1 enzyme, 100 μM NADPH, and the test compound in phosphate-buffered saline (0.1 M, pH 7.4). After preincubation at 25 °C for 15 min, the reaction was initiated by the addition of P3A as the substrate. Absorbance at 340 nm was recorded for 10 min at 25 °C. Percentage inhibition was calculated relative to a solvent control containing methanol only. Half-maximal inhibitory concentration (IC_50_) values were determined from seven-point dose–response curves generated using 2-fold serial dilution and fitted to a simple logistic model. All experiments were performed independently at least three times (*n* ≥ 3). Then data were reported as the mean ± standard error of the mean (SEM).

### 5.8 Thermal Shift Assay (TSA)

Thermal stability measurements were taken using a real-time PCR system (QuantStudio 1; Thermo Fisher Scientific Inc.) as described earlier.^42^ Each 20 μL reaction mixture contained 0.2 mg/mL AKR1B10, 25 μM test compound, 100 μM NADP^+^, and 4× SYPRO Orange dye in PBS (pH 7.4). Samples were preincubated at 25 °C for 15 min, followed by a temperature ramp from 25.0 to 99.9 °C at a rate of 0.05 °C/s. The melting temperature (*T*_m_) was found from the first derivative of the fluorescence intensity curve.

## Supplementary Material



## Data Availability

Data and Software Availability: Boltz-2 input (*.yaml) and output (*.cif, *.json, and *.npz) files of 40 (AICE001–AICE040) compounds and 132 known inhibitors from the ChEMBL database are available at https://github.com/ktomii/AKR1B10.

## Notes

Distribution of Boltz-2 prediction scores for the 867 primary candidates, colored by cluster IDs (Figure S1); pairwise similarity heatmaps of Morgan2 (ECFP4) and extended reduced graph (ErG) fingerprints for the 40 selected AICE compounds (Figure S2); chemical structures of the seven submicromolar AICE inhibitors with their AKR1B10 IC_50_ values (Figure S3); thermal shift assay (Δ*T*_m_) results for the 40 AICE compounds and their relationships with the Boltz-2 predicted affinity and binding likelihood (Figure S4); Tversky similarity between the 40 AICE compounds and the 132 known AKR1B10 inhibitors using the Morgan2 fingerprint (Figure S5) and using the ErG fingerprint (Figure S6); cluster-size stratification of the prospective hit rate (Figure S7); cluster-independent nearest-neighbor density check (Figure S8); hit-anchored hierarchical clustering dendrogram of the seven submicromolar AICE anchors and their nearest neighbors (Figure S9); per-anchor Tanimoto similarity distributions against the Enamine Discovery Diversity Set (Figure S10); Boltz-2 confidence metrics plotted against TSA Δ*T*_m_ for the 40 AICE compounds (Figure S11); Boltz-2 confidence metrics plotted against p*K*_*i*_ for the 132 known inhibitors (Figure S12); Boltz-2 prediction versus experiment for the five leakage-free Sun 2024 actives (Figure S13); an example Boltz-2 input file in YAML format (Figure S14); Boltz-2 affinity prediction for the 40 AICE compounds under use_potentials = ON versus OFF (Figure S15); Boltz-2 predicted IC_50_ under use_potentials = ON versus OFF compared with experimental end points (Figure S16); the fractions of the 132 known inhibitors present in the Boltz-2 training data (Table S1); the cluster number and cluster size of each of the 40 AICE compounds (Table S2); a threshold-sensitivity analysis of the number of compounds passing affinity_probability_binary ≥ t in each data set (Table S3); the physical feasibility (steric-clash and intraligand overlap checks) of the predicted poses (Table S4); and the AICE numbers and SMILES strings of the 40 tested compounds (Table S5) (PDF)

## Abbreviations

ABCI: AI Bridging Cloud Infrastructure
AICE: AKR1B10 Inhibitor Candidate Enrichment
AKR1B1: aldo-keto reductase 1B1
AKR1B10: aldo-keto reductase 1B10
DUD-E: Directory of Useful Decoys, Enhanced
EF: enrichment factor
ErG: extended reduced graph
FEP: free-energy perturbation
**IC**_**50**_: half-maximal inhibitory concentration
**NADP** ^**+**^: nicotinamide adenine dinucleotide phosphate (oxidized)
NADPH: nicotinamide adenine dinucleotide phosphate (reduced)
P3A: pyridine-3-aldehyde
PBS: phosphate-buffered saline
ROC-AUC: area under the receiver operating characteristic curve
SEM: standard error of the mean
***T***_**m**_: melting temperature
TSA: thermal shift assay

## References

[1] Corso G., Stärk H., Jing B., Barzilay R., Jaakkola T. (2022). DiffDock:
Diffusion Steps, Twists, and Turns for Molecular Docking. arXiv.

[2] I.., Labs : Alphafold 3 predicts the structure and interactions of all of life’s molecules, 2024. https://www.isomorphiclabs.com/articles/alphafold-3-predicts-the-structure-and-interactions-of-all-of-lifes-molecules. (Accessed 15 March 2026).

[3] Friesner R.
A., Banks J. L., Murphy R. B., Halgren T. A., Klicic J. J., Mainz D. T., Repasky M. P., Knoll E. H., Shelley M., Perry J. K., Shaw D. E., Francis P., Shenkin P. S. G. (2004). A New
Approach for Rapid, Accurate Docking and Scoring. 1. Method and Assessment
of Docking Accuracy. J. Med. Chem..

[4] Trott O., Olson A. J. (2010). AutoDock Vina: improving
the speed and accuracy of
docking with a new scoring function, efficient optimization, and multithreading. J. Comput. Chem..

[5] Abramson J., Adler J., Dunger J., Evans R., Green T., Pritzel A., Ronneberger O., Willmore L., Ballard A. J., Bambrick J., Bodenstein S. W., Evans D. A., Hung C.-C., O’Neill M., Reiman D., Tunyasuvunakool K., Wu Z., Žemgulytė A., Arvaniti E., Beattie C., Bertolli O., Bridgland A., Cherepanov A., Congreve M., Cowen-Rivers A. I., Cowie A., Figurnov M., Fuchs F. B., Gladman H., Jain R., Khan Y. A., Low C. M. R., Perlin K., Potapenko A., Savy P., Singh S., Stecula A., Thillaisundaram A., Tong C., Yakneen S., Zhong E. D., Zielinski M., Žídek A., Bapst V., Kohli P., Jaderberg M., Hassabis D., Jumper J. M. (2024). Accurate structure prediction of
biomolecular interactions with AlphaFold 3. Nature.

[6] Passaro S., Corso G., Wohlwend J., Reveiz M., Thaler S., Somnath V. R., Getz N., Portnoi T., Roy J., Stark H., Kwabi-Addo D., Beaini D., Jaakkola T., Barzilay R. (2025). Boltz-2: Towards Accurate
and Efficient Binding Affinity
Prediction. bioRxiv.

[7] Wohlwend J., Corso G., Passaro S., Getz N., Reveiz M., Leidal K., Swiderski W., Atkinson L., Portnoi T., Chinn I., Silterra J., Jaakkola T., Barzilay R. (2025). Boltz-1 Democratizing
Biomolecular Interaction Modeling. bioRxiv.

[8] Bryant P., Kelkar A., Guljas A., Clementi C., Noé F. (2024). Structure
prediction of protein-ligand complexes from sequence information with
Umol. Nat. Commun..

[9] Krishna R., Wang J., Ahern W., Sturmfels P., Venkatesh P., Kalvet I., Lee G. R., Morey-Burrows F. S., Anishchenko I., Humphreys I. R., McHugh R., Vafeados D., Li X., Sutherland G. A., Hitchcock A., Hunter C. N., Kang A., Brackenbrough E., Bera A. K., Baek M., DiMaio F., Baker D. (2024). Generalized
biomolecular modeling and design with RoseTTAFold All-Atom. Science.

[10] Jumper J., Evans R., Pritzel A., Green T., Figurnov M., Ronneberger O., Tunyasuvunakool K., Bates R., Žídek A., Potapenko A., Bridgland A., Meyer C., Kohl S. A. A., Ballard A. J., Cowie A., Romera-Paredes B., Nikolov S., Jain R., Adler J., Back T., Petersen S., Reiman D., Clancy E., Zielinski M., Steinegger M., Pacholska M., Berghammer T., Bodenstein S., Silver D., Vinyals O., Senior A. W., Kavukcuoglu K., Kohli P., Hassabis D. (2021). Highly accurate protein
structure prediction with AlphaFold. Nature.

[11] Sherman W., Day T., Jacobson M. P., Friesner R. A., Farid R. (2006). Novel procedure for
modeling ligand/receptor induced fit effects. J. Med. Chem..

[12] Škrinjar P., Eberhardt J., Tauriello G., Schwede T., Durairaj J. (2025). Have protein-ligand
cofolding methods moved beyond memorisation?. bioRxiv.

[13] Bret G., Sindt F., Rognan D. (2026). Assessing
Boltz-2 Performance for
the Binding Classification of Docking Hits. J. Chem. Inf. Model..

[14] Ma J., Yan R., Zu X., Cheng J. M., Rao K., Liao D. F., Cao D. (2008). Aldo-keto reductase family 1 B10
affects fatty acid synthesis by
regulating the stability of acetyl-CoA carboxylase-alpha in breast
cancer cells. J. Biol. Chem..

[15] Matsunaga T., Wada Y., Endo S., Soda M., El-Kabbani O., Hara A. (2012). Aldo-Keto Reductase 1B10 and Its
Role in Proliferation Capacity of
Drug-Resistant Cancers. Front. Pharmacol..

[16] Ye X., Li C., Zu X., Lin M., Liu Q., Liu J., Xu G., Chen Z., Xu Y., Liu L., Luo D., Cao Z., Shi G., Feng Z., Deng H., Liao Q., Cai C., Liao D. F., Wang J., Jin J., Cao D. (2019). A Large-Scale
Multicenter Study Validates Aldo-Keto Reductase Family 1 Member B10
as a Prevalent Serum Marker for Detection of Hepatocellular Carcinoma. Hepatology.

[17] Endo S., Xia S., Suyama M., Morikawa Y., Oguri H., Hu D., Ao Y., Takahara S., Horino Y., Hayakawa Y., Watanabe Y., Gouda H., Hara A., Kuwata K., Toyooka N., Matsunaga T., Ikari A. (2017). Synthesis of Potent and Selective
Inhibitors of Aldo-Keto Reductase 1B10 and Their Efficacy against
Proliferation, Metastasis, and Cisplatin Resistance of Lung Cancer
Cells. J. Med. Chem..

[18] Ko H. H., Chou H. Y. E., Hou H. H., Kuo W. T., Liu W. W., Yen-Ping Kuo M., Cheng S. J. (2025). Oleanolic acid inhibits aldo-keto
reductase family 1 member B10-induced cancer stemness and avoids cisplatin-based
chemotherapy resistance via the Snail signaling pathway in oral squamous
cell carcinoma cell lines. J. Dent Sci..

[19] Kikuya M., Furuichi K., Hirao T., Endo S., Toyooka N., Ito K., Aoki S. (2021). Aldo-keto
reductase inhibitors increase the anticancer
effects of tyrosine kinase inhibitors in chronic myelogenous leukemia. J. Pharmacol Sci..

[20] Matsunaga T., Suzuki A., Kezuka C., Okumura N., Iguchi K., Inoue I., Soda M., Endo S., El-Kabbani O., Hara A., Ikari A. (2016). Aldo-keto reductase
1B10 promotes
development of cisplatin resistance in gastrointestinal cancer cells
through down-regulating peroxisome proliferator-activated receptor-gamma-dependent
mechanism. Chem. Biol. Interact.

[21] Morikawa Y., Kezuka C., Endo S., Ikari A., Soda M., Yamamura K., Toyooka N., El-Kabbani O., Hara A., Matsunaga T. (2015). Acquisition
of doxorubicin resistance
facilitates migrating and invasive potentials of gastric cancer MKN45
cells through up-regulating aldo-keto reductase 1B10. Chem. Biol. Interact.

[22] Hara A., Endo S., Matsunaga T., Soda M., El-Kabbani O., Yashiro K. (2016). Inhibition of aldo-keto
reductase family 1 member B10
by unsaturated fatty acids. Arch. Biochem. Biophys..

[23] Soda M., Endo S., Matsunaga T., Zhao H. T., El-Kabbani O., Iinuma M., Yamamura K., Hara A. (2012). Inhibition of human
aldose reductase-like protein (AKR1B10) by alpha- and gamma-mangostins,
major components of pericarps of mangosteen. Biol. Pharm. Bull..

[24] Takemura M., Endo S., Matsunaga T., Soda M., Zhao H. T., El-Kabbani O., Tajima K., Iinuma M., Hara A. (2011). Selective
inhibition of the tumor marker aldo-keto reductase family member 1B10
by oleanolic acid. J. Nat. Prod..

[25] Matsunaga T., Endo S., Soda M., Zhao H. T., El-Kabbani O., Tajima K., Hara A. (2009). Potent and
selective inhibition of
the tumor marker AKR1B10 by bisdemethoxycurcumin: probing the active
site of the enzyme with molecular modeling and site-directed mutagenesis. Biochem. Biophys. Res. Commun..

[26] Endo S., Matsunaga T., Mamiya H., Ohta C., Soda M., Kitade Y., Tajima K., Zhao H. T., El-Kabbani O., Hara A. (2009). Kinetic studies of AKR1B10, human
aldose reductase-like protein:
endogenous substrates and inhibition by steroids. Arch. Biochem. Biophys..

[27] Endo S., Hu D., Suyama M., Matsunaga T., Sugimoto K., Matsuya Y., El-Kabbani O., Kuwata K., Hara A., Kitade Y., Toyooka N. (2013). Synthesis
and structure-activity relationship of 2-phenyliminochromene
derivatives as inhibitors for aldo-keto reductase (AKR) 1B10. Bioorg. Med. Chem..

[28] Endo S., Matsunaga T., Kuwata K., Zhao H.-T., El-Kabbani O., Kitade Y., Hara A. (2010). Chromene-3-carboxamide
derivatives
discovered from virtual screening as potent inhibitors of the tumour
marker, AKR1B10. Bioorg. Med. Chem..

[29] Soda M., Hu D., Endo S., Takemura M., Li J., Wada R., Ifuku S., Zhao H. T., El-Kabbani O., Ohta S., Yamamura K., Toyooka N., Hara A., Matsunaga T. (2012). Design, synthesis
and evaluation of caffeic acid phenethyl
ester-based inhibitors targeting a selectivity pocket in the active
site of human aldo-keto reductase 1B10. Eur.
J. Med. Chem..

[30] Mysinger M. M., Carchia M., Irwin J. J., Shoichet B. K. (2012). Directory of Useful
Decoys, Enhanced (DUD-E): Better Ligands and Decoys for Better Benchmarking. J. Med. Chem..

[31] Zdrazil B., Felix E., Hunter F., Manners E. J., Blackshaw J., Corbett S., de Veij M., Ioannidis H., Lopez D. M., Mosquera J. F., Magarinos M. P., Bosc N., Arcila R., Kizilören T., Gaulton A., Bento A. P., Adasme M. F., Monecke P., Landrum G. A., Leach A. R. (2024). The ChEMBL Database in 2023: a drug
discovery platform spanning multiple bioactivity data types and time
periods. Nucleic Acids Res..

[32] Gallego O., Ruiz F. X., Ardèvol A., Domínguez M., Alvarez R., de Lera A. R., Rovira C., Farrés J., Fita I., Parés X. (2007). Structural
basis for the high all-trans-retinaldehyde
reductase activity of the tumor marker AKR1B10. Proc. Natl. Acad. Sci. U. S. A..

[33] Sotriffer C. A., Krämer O., Klebe G. (2004). Probing flexibility
and ″induced-fit″
phenomena in aldose reductase by comparative crystal structure analysis
and molecular dynamics simulations. Proteins.

[34] Klebe G., Krämer O., Sotriffer C. (2004). Strategies for the design of inhibitors
of aldose reductase, an enzyme showing pronounced induced-fit adaptations. Cellular and Molecular Life Sciences CMLS.

[35] Truchon J.-F., Bayly C. I. (2007). Evaluating Virtual
Screening Methods: Good and Bad
Metrics for the “Early Recognition” Problem. J. Chem. Inf. Model..

[36] Esposito C., Landrum G. A., Schneider N., Stiefl N., Riniker S. (2021). GHOST: Adjusting
the Decision Threshold to Handle Imbalanced Data in Machine Learning. J. Chem. Inf. Model..

[37] Buttenschoen M., Morris G. M., Deane C. M. (2024). PoseBusters: AI-based
docking methods
fail to generate physically valid poses or generalise to novel sequences. Chem. Sci..

[38] Stiefl N., Watson I. A., Baumann K., Zaliani A. (2006). ErG: 2D Pharmacophore
Descriptions for Scaffold Hopping. J. Chem.
Inf. Model..

[39] Matter H. (1997). Selecting
optimally diverse compounds from structure databases: a validation
study of two-dimensional and three-dimensional molecular descriptors. J. Med. Chem..

[40] Wassermann A. M., Camargo L. M., Auld D. S. (2014). Composition and applications of focus
libraries to phenotypic assays. Front. Pharmacol..

[41] Zhu T., Cao S., Su P. C., Patel R., Shah D., Chokshi H. B., Szukala R., Johnson M. E., Hevener K. E. (2013). Hit identification
and optimization in virtual screening: practical recommendations based
on a critical literature analysis. J. Med. Chem..

[42] Kabir A., Endo S., Toyooka N., Fukuoka M., Kuwata K., Kamatari Y. O. (2017). Evaluation of compound
selectivity of aldo-keto reductases
using differential scanning fluorimetry. J.
Biochem..

[43] Niesen F. H., Berglund H., Vedadi M. (2007). The use of differential scanning
fluorimetry to detect ligand interactions that promote protein stability. Nat. Protoc..

[44] Cimmperman P., Baranauskiene L., Jachimoviciu̅te S., Jachno J., Torresan J., Michailoviene V., Matuliene J., Sereikaite J., Bumelis V., Matulis D. (2008). A quantitative model
of thermal stabilization and destabilization of proteins by ligands. Biophys. J..

[45] Bajusz D., Rácz A., Héberger K. (2015). Why is Tanimoto
index an appropriate
choice for fingerprint-based similarity calculations?. J. Cheminform.

[46] Rogers D., Hahn M. (2010). Extended-Connectivity
Fingerprints. J. Chem.
Inf. Model..

[47] Zhang L., Zhang H., Zhao Y., Li Z., Chen S., Zhai J., Chen Y., Xie W., Wang Z., Li Q., Zheng X., Hu X. (2013). Inhibitor
selectivity between aldo–keto
reductase superfamily members AKR1B10 and AKR1B1: Role of Trp112 (Trp111). FEBS Lett..

[48] Di
Costanzo L., Penning T. M., Christianson D. W. (2009). Aldo-keto
reductases in which the conserved catalytic histidine is substituted. Chem. Biol. Interact.

[49] Endo S., Matsunaga T., Nishinaka T. (2021). The Role of
AKR1B10 in Physiology
and Pathophysiology. Metabolites.

[50] Pagadala N. S., Syed K., Tuszynski J. (2017). Software for molecular docking: a
review. Biophysical Reviews.

[51] Irwin J. J., Shoichet B. K. (2016). Docking Screens
for Novel Ligands Conferring New Biology. J.
Med. Chem..

[52] Li Y., Han L., Liu Z., Wang R. (2014). Comparative assessment of scoring
functions on an updated benchmark: 2. Evaluation methods and general
results. J. Chem. Inf. Model..

[53] Su M., Yang Q., Du Y., Feng G., Liu Z., Li Y., Wang R. (2019). Comparative
Assessment of Scoring Functions: The CASF-2016
Update. J. Chem. Inf. Model..

[54] Warren G. L., Andrews C. W., Capelli A. M., Clarke B., LaLonde J., Lambert M. H., Lindvall M., Nevins N., Semus S. F., Senger S., Tedesco G., Wall I. D., Woolven J. M., Peishoff C. E., Head M. S. (2006). A critical assessment
of docking
programs and scoring functions. J. Med. Chem..

[55] Pantsar T., Poso A. (2018). Binding Affinity via
Docking: Fact and Fiction. Molecules.

[56] Olsson T. S., Ladbury J. E., Pitt W. R., Williams M. A. (2011). Extent of enthalpy–entropy
compensation in protein-ligand interactions. Protein Sci..

[57] Sun N., Ma S., Jin L., Wang Y., Zhou C., Zhang X., Kang H., Zhou M., Yang H., Shu P. (2024). Development
of AKR1B10 inhibitors from *Ajuga nipponensis* based
on diseases and targets. Fitoterapia.

[58] Steinegger M., Söding J. (2017). MMseqs2 enables sensitive protein
sequence searching
for the analysis of massive data sets. Nat.
Biotechnol..

[59] Jacobsson M., Lidén P., Stjernschantz E., Boström H., Norinder U. (2003). Improving Structure-Based
Virtual Screening by Multivariate
Analysis of Scoring Data. J. Med. Chem..

[60] Fawcett T. (2006). An introduction
to ROC analysis. Pattern Recognition Letters.

[61] Rousseeuw P. J. (1987). Silhouettes:
A graphical aid to the interpretation and validation of cluster analysis. Journal of Computational and Applied Mathematics.

[62] Swamidass S. J., Baldi P. (2007). Bounds and Algorithms for Fast Exact
Searches of Chemical Fingerprints
in Linear and Sublinear Time. J. Chem. Inf.
Model..

