# Supplementary material for: Efficient Discovery of Potent AKR1B10 Inhibitors Using Co-folding Model Boltz‑2
Source: J Chem Inf Model. 2026 Sep 12;66(18):12100–14. doi: 10.1021/acs.jcim.6c00874 (PMC13625949; doi:10.1021/acs.jcim.6c00874)
Supplement: Supplementary file 1 [file ci6c00874_si_001.pdf]

Supporting Information

Efficient Discovery of Potent AKR1B10 Inhibitors Using Co-folding Model Boltz-2

Shinya Kawano[a,b]*, Akira Ikari*[a]*, Satoshi Endo*[c,d,e*]*, and Kentaro Tomii* [b*]

[a] *Laboratory of Biochemistry, Gifu Pharmaceutical University, Gifu, Gifu 501-1196, Japan*

[b] *Artificial Intelligence Research Center (AIRC), National Institute of Advanced Industrial Science and Technology (AIST), Koto-ku, Tokyo 135-0064, Japan*

[c] *United Graduate School of Drug Discovery and Medical Information Sciences, Gifu University, Gifu, Gifu 501-1194, Japan*

[d] *Center for One Medicine Innovative Translational Research (COMIT), Gifu University, Gifu, Gifu 501-1193, Japan*

[e] *Preemptive Food Research Center, Gifu University, Gifu, 501-1193, Japan*

* E-mail: endou.satoshi.n1@f.gifu-u.ac.jp, k-tomii@aist.go.jp

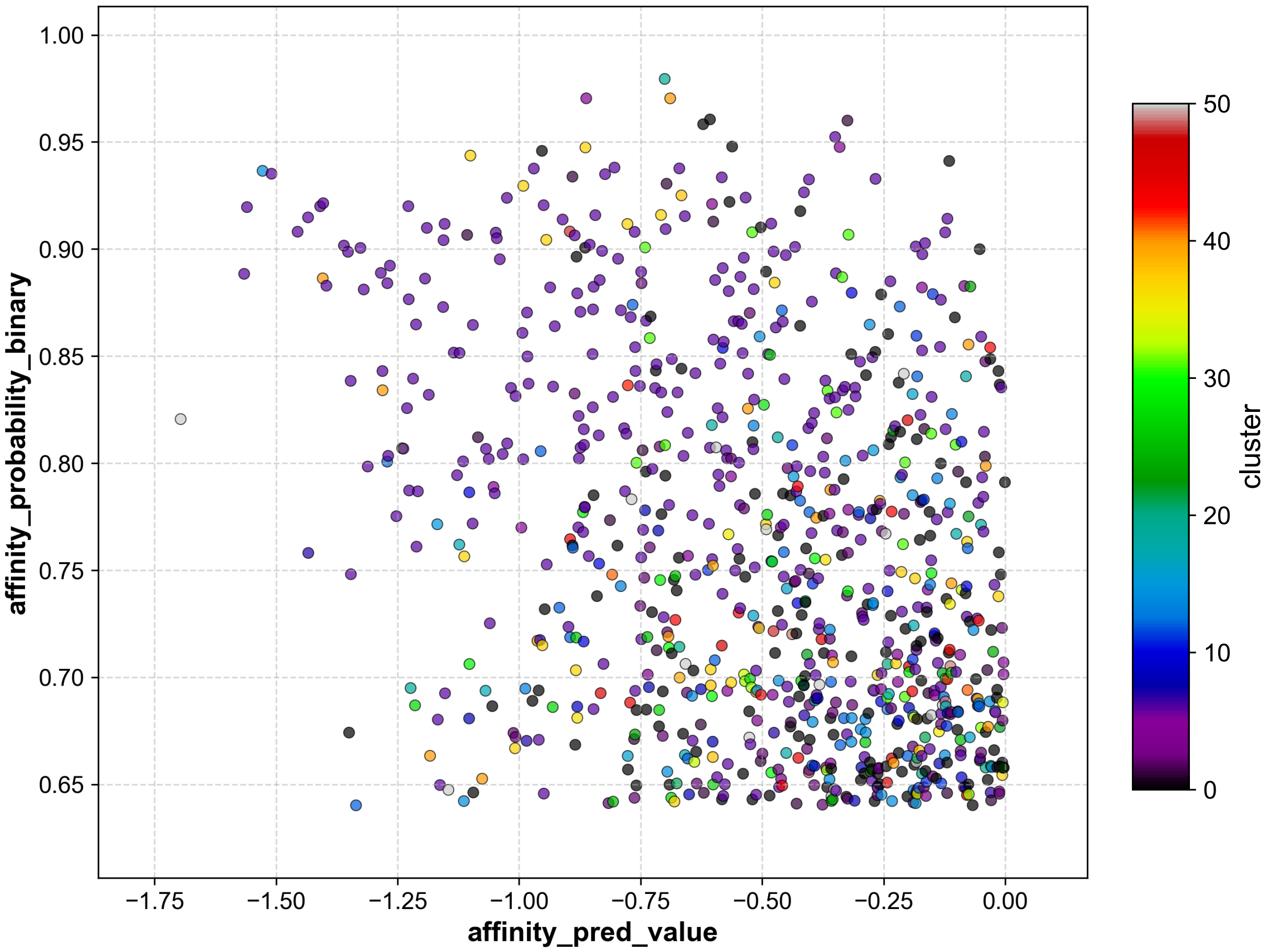

Fig. S1 Distribution of Boltz-2 prediction scores for the 867 primary candidates. Scatter plot of affinity_pred_value (x-axis) against affinity_probability_binary (y-axis) for the 867 compounds from the Enamine Discovery Diversity Set that exceeded the prospective thresholds (affinity_probability_binary ≥ 0.64 and affinity_pred_value ≤ 0). Points are colored by cluster ID (0–50) from the hierarchical agglomerative clustering (*k* = 51) used for diversity-based selection.

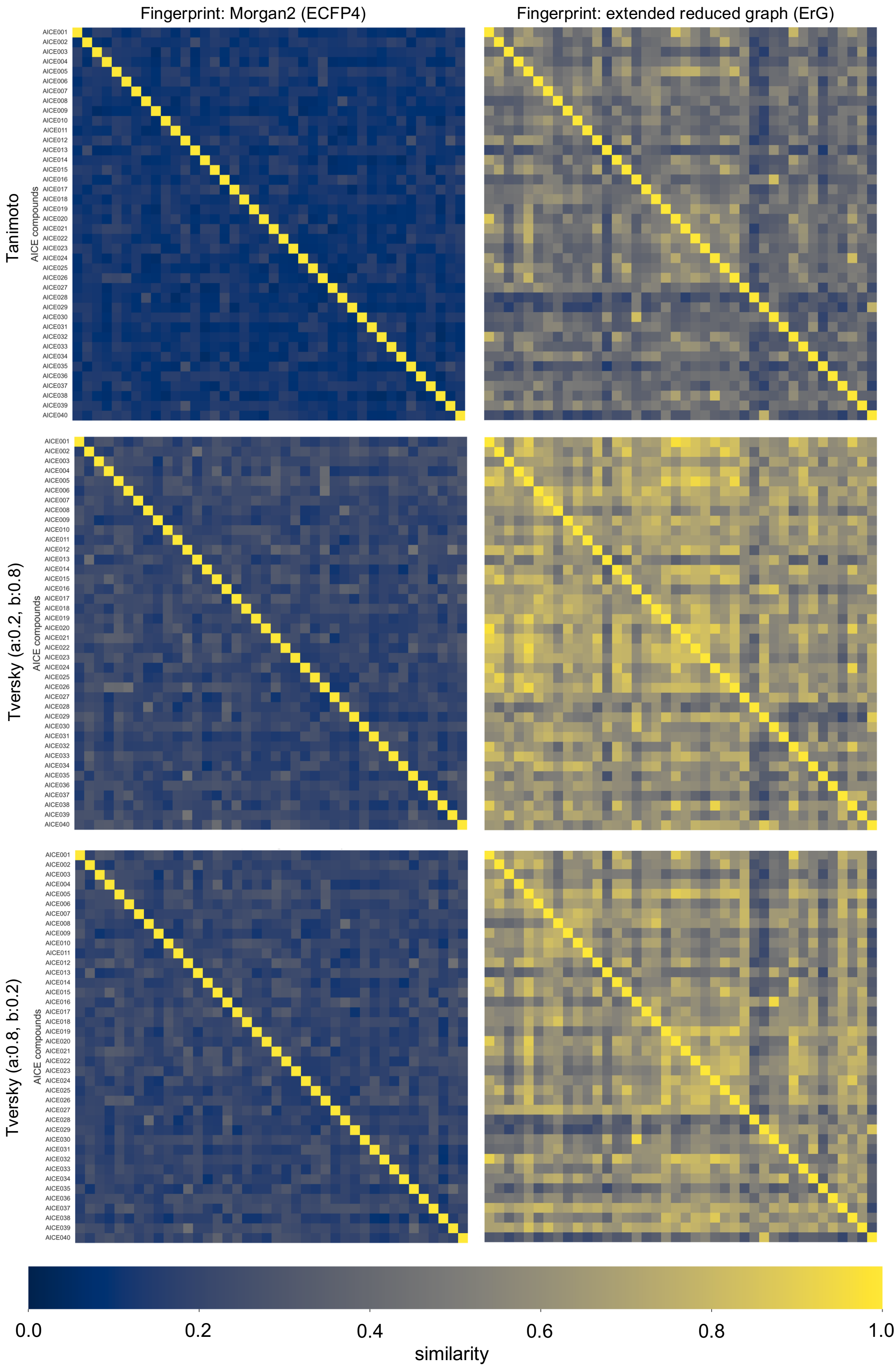

Fingerprint: Morgan2 (ECFP4)
Fingerprint: extended reduced graph (ErG)
Tanimoto
Tversky (a:0.2, b:0.8)
Tversky (a:0.8, b:0.2)
AICE compounds
AICE001
AICE002
AICE003
AICE004
AICE005
AICE006
AICE007
AICE008
AICE009
AICE010
AICE011
AICE012
AICE013
AICE014
AICE015
AICE016
AICE017
AICE018
AICE019
AICE020
AICE021
AICE022
AICE023
AICE024
AICE025
AICE026
AICE027
AICE028
AICE029
AICE030
AICE031
AICE032
AICE033
AICE034
AICE035
AICE036
AICE037
AICE038
AICE039
AICE040
0.0
0.2
0.4
0.6
0.8
1.0
similarity

Fig. S2 Pairwise similarity among the 40 selected AICE compounds. Heatmaps of pairwise similarity computed with Morgan2 (ECFP4; left column) and extended reduced graph (ErG; right column) fingerprints. Rows show the Tanimoto coefficient (top), the Tversky index with $\alpha = 0.2$, $\beta = 0.8$ (middle), and the Tversky index with $\alpha = 0.8$, $\beta = 0.2$ (bottom). The color scale represents similarity from 0.0 (blue) to 1.0 (yellow).

AICE001

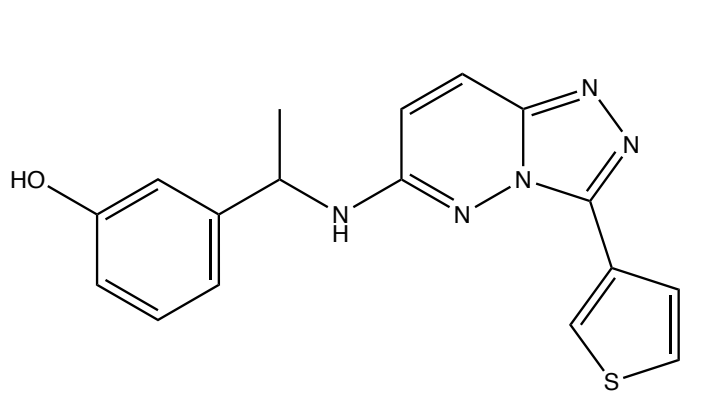

0.717 ± 0.050 μM

AICE003

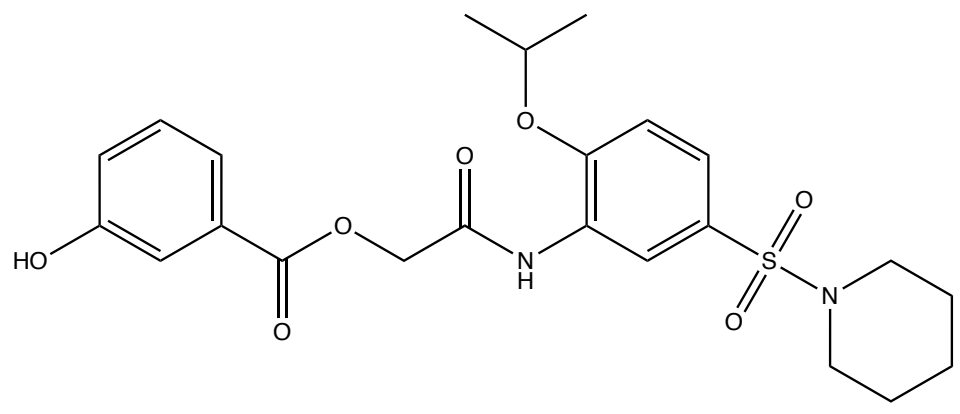

0.197 ± 0.012 μM

AICE023

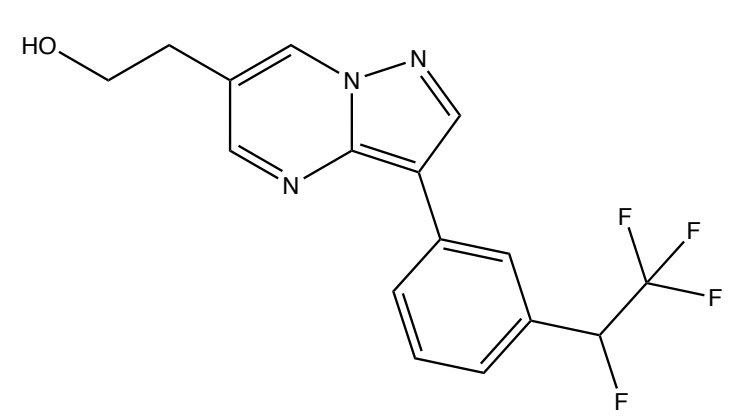

0.325 ± 0.040 μM

AICE024

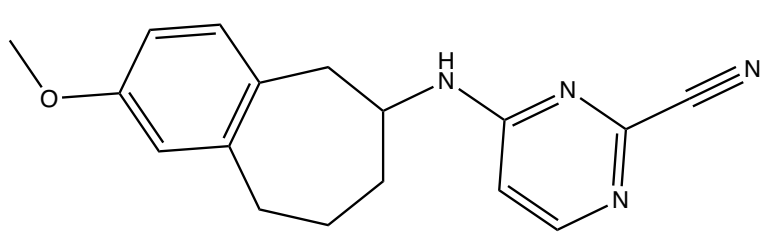

0.743 ± 0.073 μM

AICE032

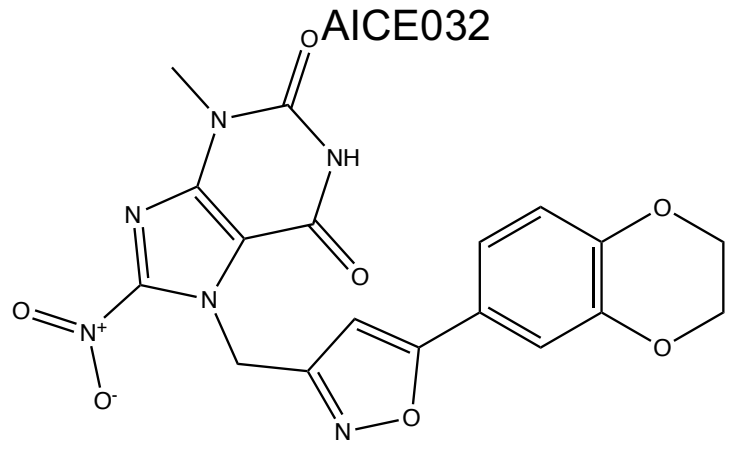

0.604 ± 0.042 μM

AICE036

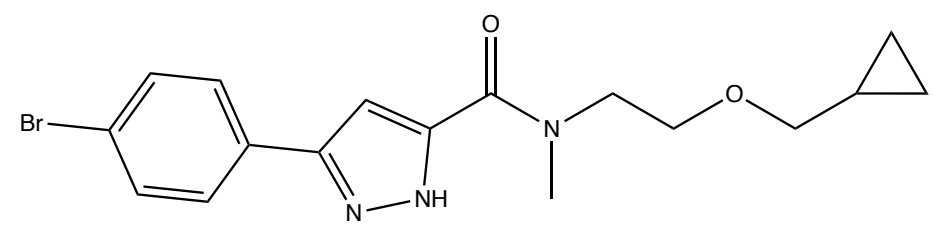

0.298 ± 0.045 μM

AICE038

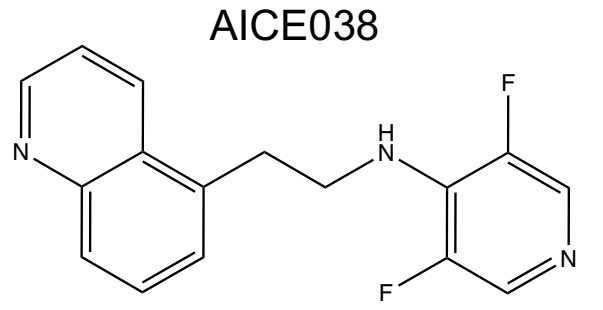

0.728 ± 0.111 μM

Fig. S3 Chemical structures of the seven AICE compounds. Two-dimensional structures of AICE001, AICE003, AICE023, AICE024, AICE032, AICE036, and AICE038, each annotated with its AKR1B10 IC_{50} value (mean ± SEM, $n \geq 3$).

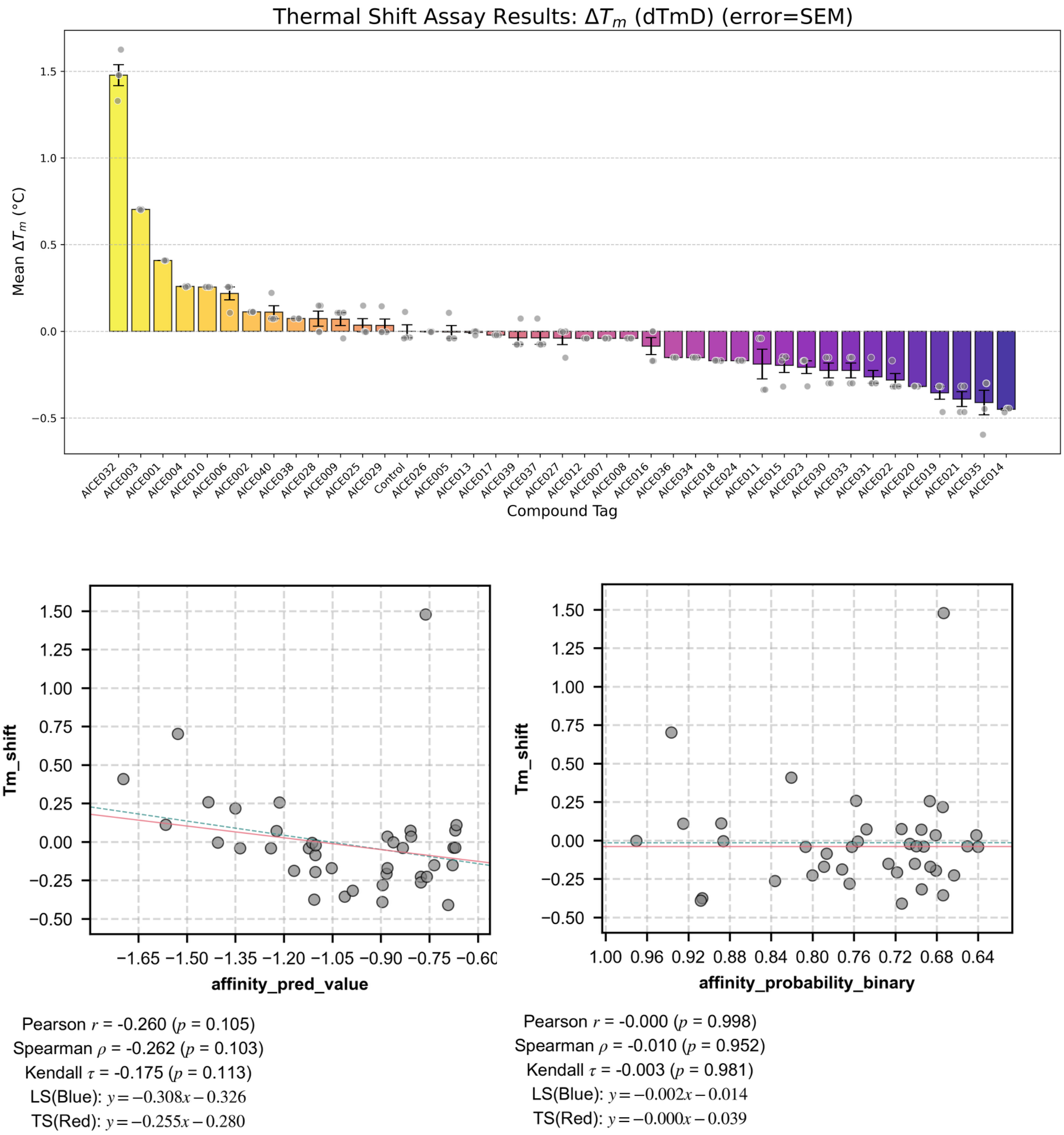

Fig. S4 Thermal shift assay (TSA) results for the 40 AICE compounds. (Top) Mean melting-temperature shift (ΔT_m, ºC) relative to the solvent control, shown in descending order; error bars denote SEM (n ≥ 3). (Bottom) Scatter plots of ΔT_m against the Boltz-2 affinity_pred_value (left) and affinity_probability_binary (right), with Pearson, Spearman, and Kendall correlation coefficients and least-squares (blue) and Theil–Sen (red) regression lines.

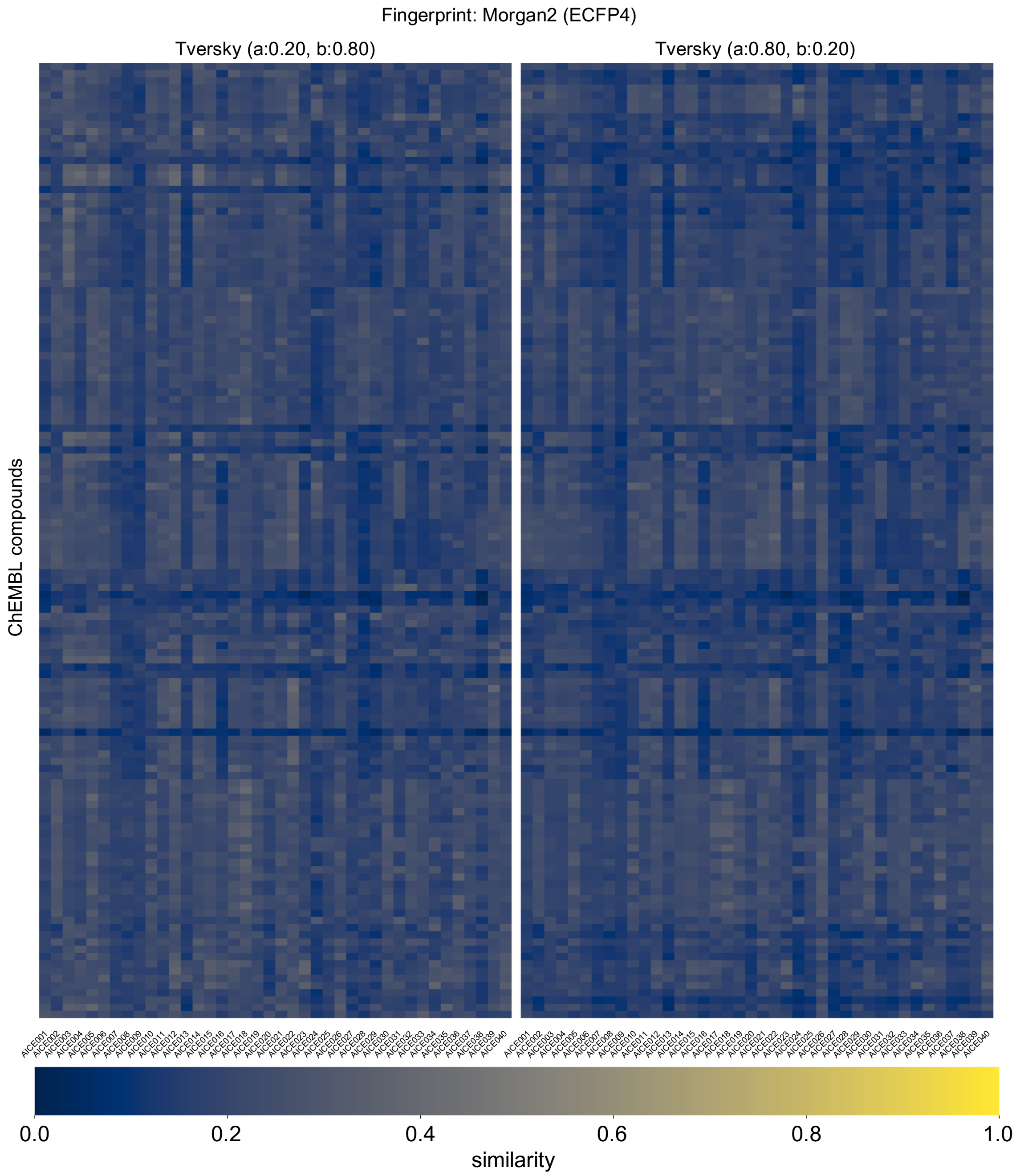

Fig. S5 Tversky similarity between the 40 AICE compounds and the 132 known AKR1B10 inhibitors (Morgan2 fingerprint). Heatmaps of the Tversky index computed with Morgan2 (ECFP4) fingerprints under asymmetric weightings α = 0.20, β = 0.80 (left) and α = 0.80, β = 0.20 (right). Rows correspond to the 132 ChEMBL inhibitors and columns to the 40 AICE compounds. The color scale represents similarity from 0.0 to 1.0.

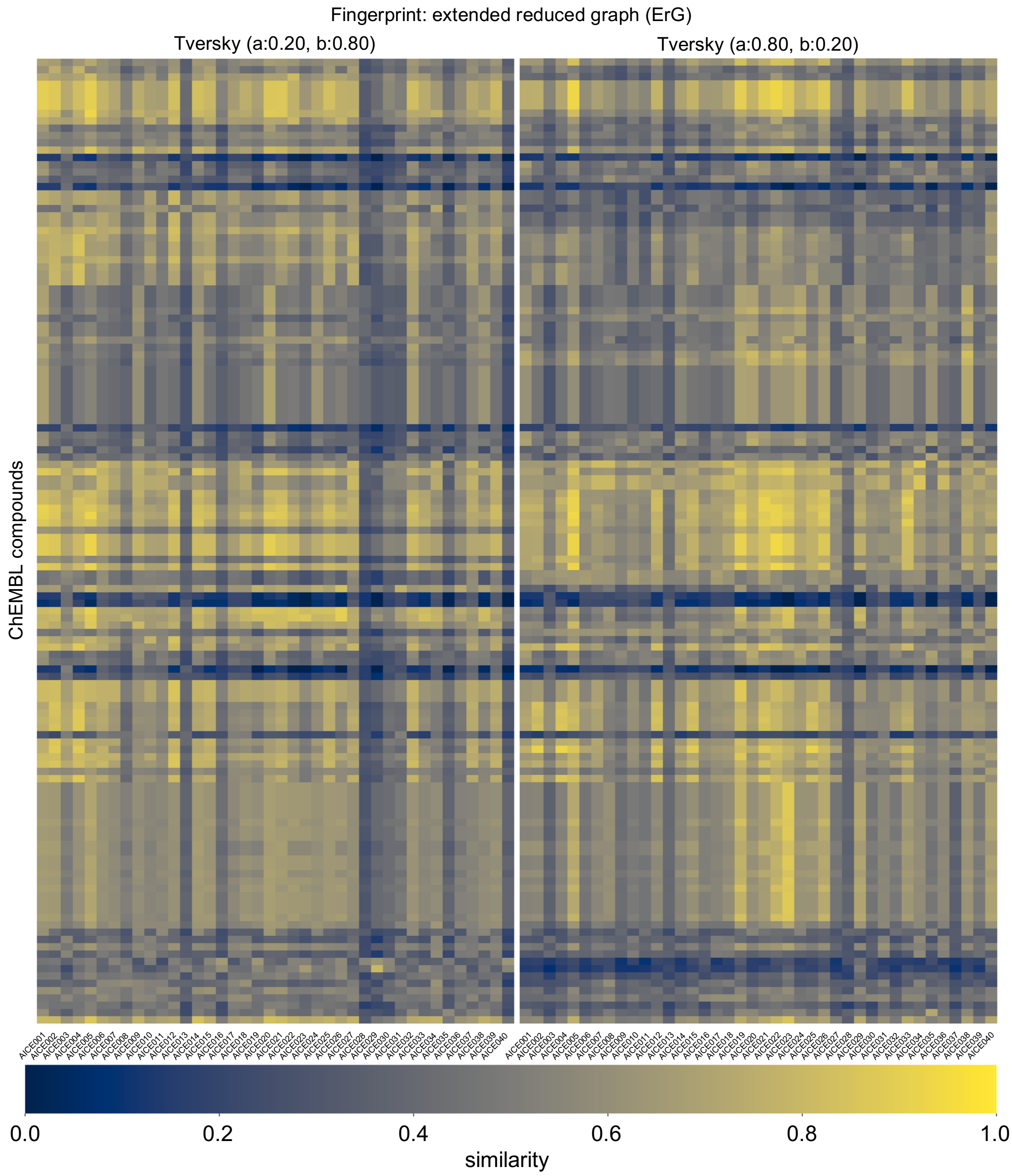

Fig. S6 Tversky similarity between the 40 AICE compounds and the 132 known AKR1B10 inhibitors (ErG fingerprint). As in Fig. S5, but using extended reduced graph (ErG) fingerprints under Tversky weightings α = 0.20, β = 0.80 (left) and α = 0.80, β = 0.20 (right).

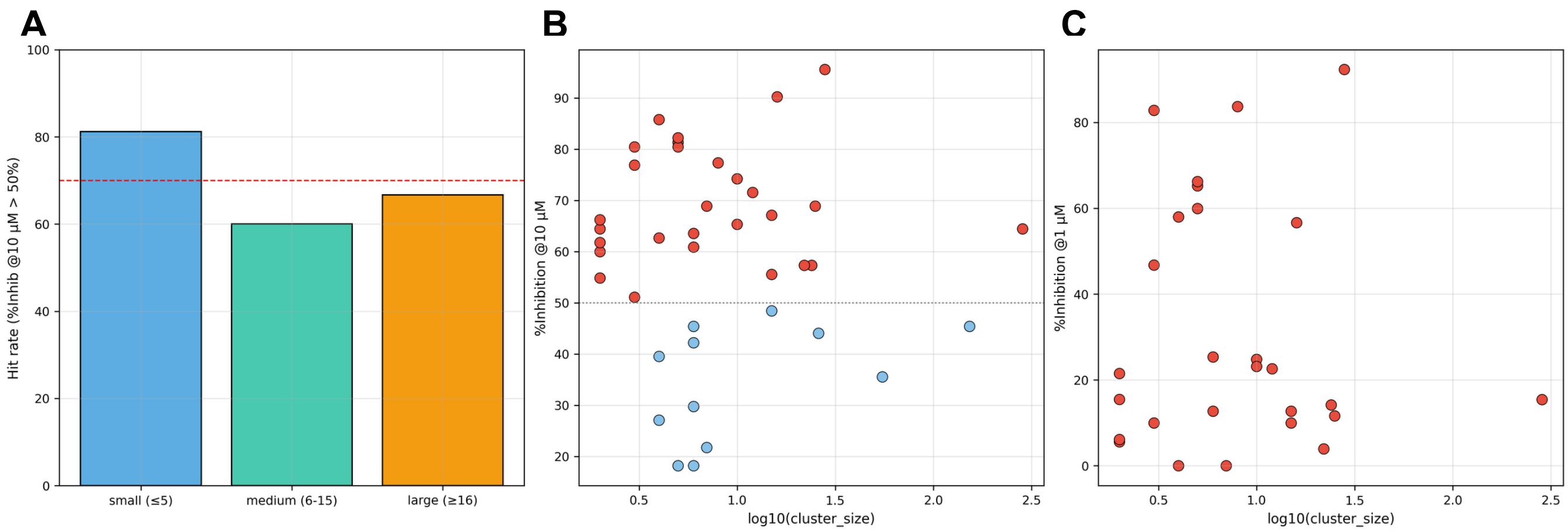

Fig. S7 Cluster-size stratification of the prospective hit rate. (A) Hit rate of the 40 tested AICE compounds, stratified by Enamine cluster size: small (≤ 5), medium (6–15), and large (≥ 16). The hit rates were 81.2%, 60.0%, and 66.7%, respectively. (B, C) Scatter plot of $\log_{10}$(cluster size) against %inhibition at 10 μM (Pearson $r = -0.020$, n = 40), showing no systematic dependence of hit rate on cluster size.

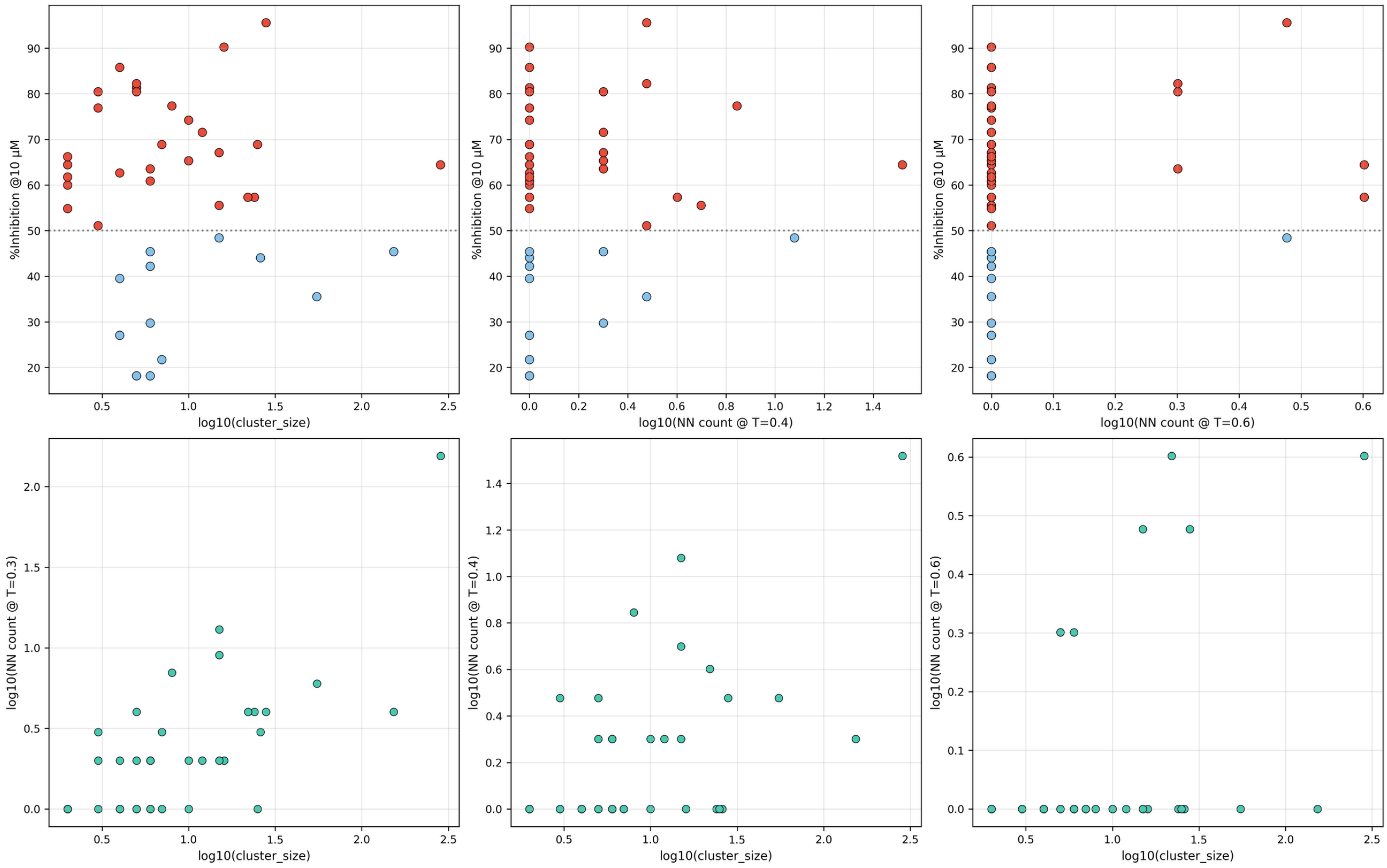

Fig. S8 Cluster-independent nearest-neighbor density check. Pearson correlation between the Tanimoto-based nearest-neighbor density (at thresholds $T = 0.3, 0.4, 0.5, 0.6$, and 0.7) and % inhibition at 10 μM for the 40 AICE compounds. $|r|$ remains below 0.30 at all five thresholds, confirming that the principal conclusion does not depend on the Morgan2/Tanimoto/$k = 51$ clustering procedure.

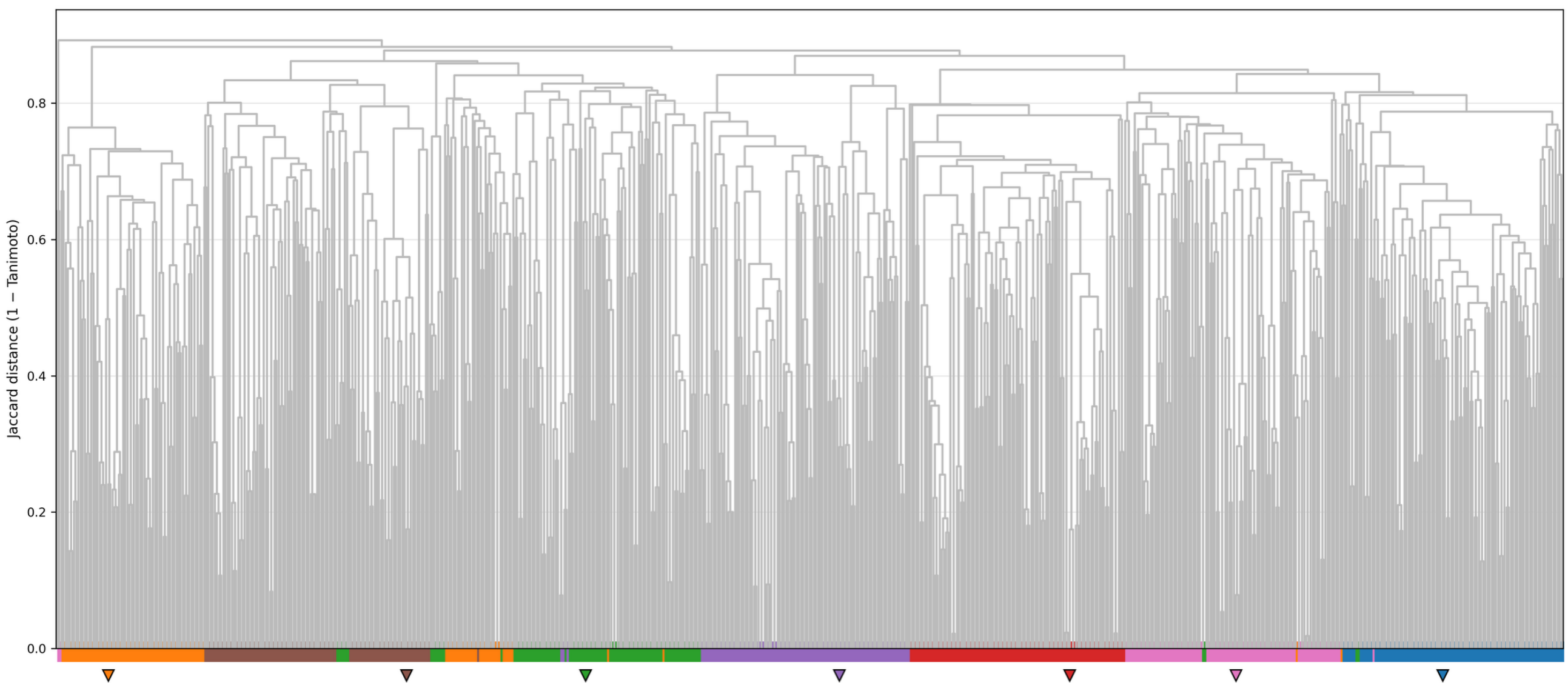

Fig. S9 Hit-anchored hierarchical clustering. Dendrogram (Morgan2-2048 Tanimoto, average linkage) of a 707-compound subset comprising the seven sub-μM AICE anchors (AICE001, 003, 023, 024, 032, 036, and 038) and their top-100 nearest neighbors from the Enamine Discovery Diversity Set. The seven anchors distribute across independent branches, indicating that the sub-μM inhibitors are not concentrated in a single chemotype family.

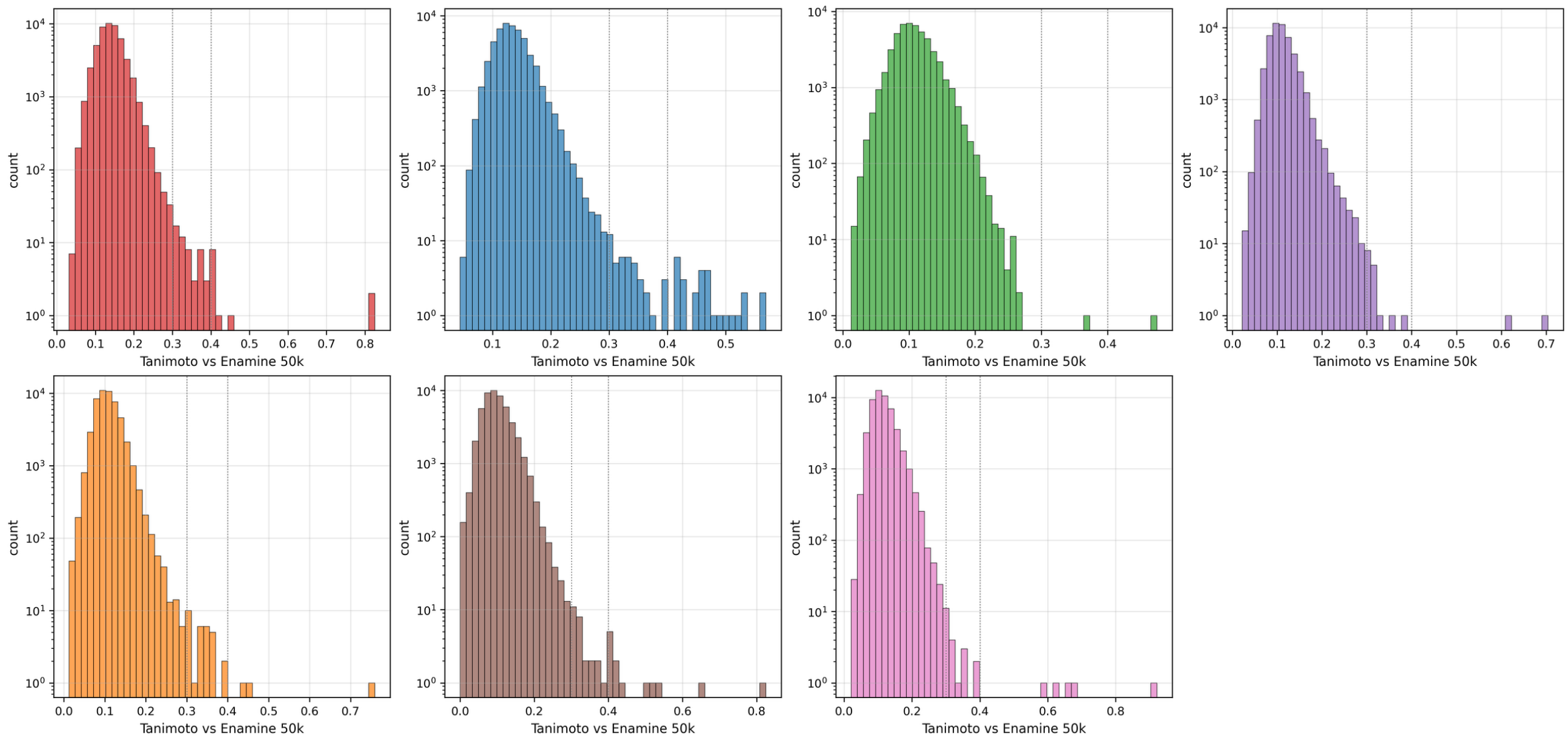

Fig. S10 Per-anchor Tanimoto distribution. Histograms of the Tanimoto similarity (Morgan2-2048) between each of the seven sub-μM AICE anchors and the full 50,240-compound Enamine Discovery Diversity Set (self-excluded; counts on a log scale). The median Tanimoto is approximately 0.10 for every anchor, indicating a low average similarity of each sub-μM inhibitor to the bulk of the library.

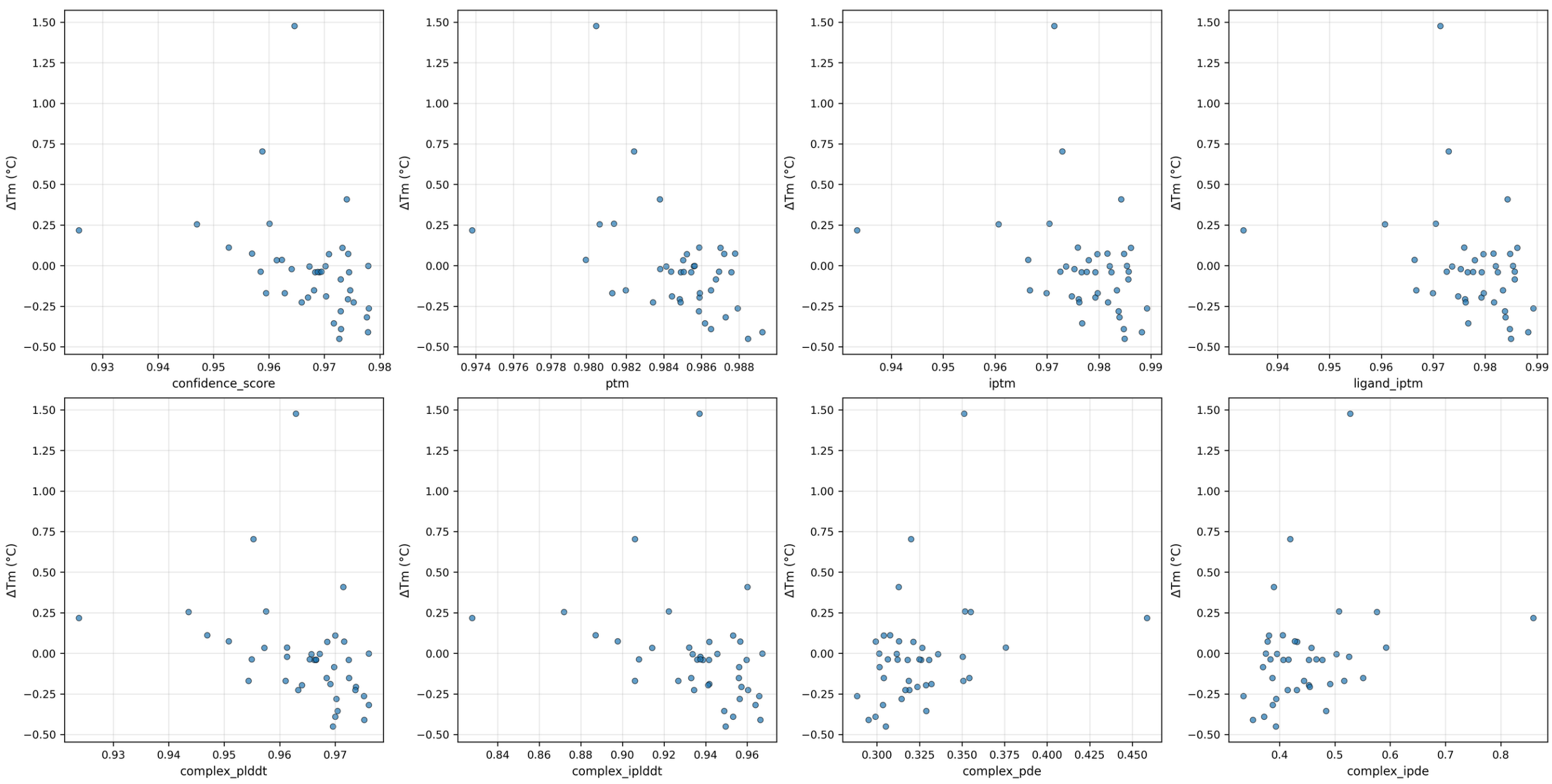

Fig. S11 Boltz-2 confidence metrics against TSA ΔT_m for the 40 AICE compounds. Scatter plots of the eight Boltz-2 confidence metrics (confidence_score, ptm, iptm, ligand_iptm, complex_plddt, complex_iplddt, complex_pde, complex_ipde) against TSA ΔT_m. ptm and complex_plddt show moderate negative correlations (Pearson r = −0.50 and Spearman ρ = −0.52, respectively); the remaining metrics do not exhibit a significant linear relationship. The same eight metrics yielded $|r| < 0.30$ against %inhibition at 10 μM and pIC_{50} on the same AICE 40 set (data not shown).

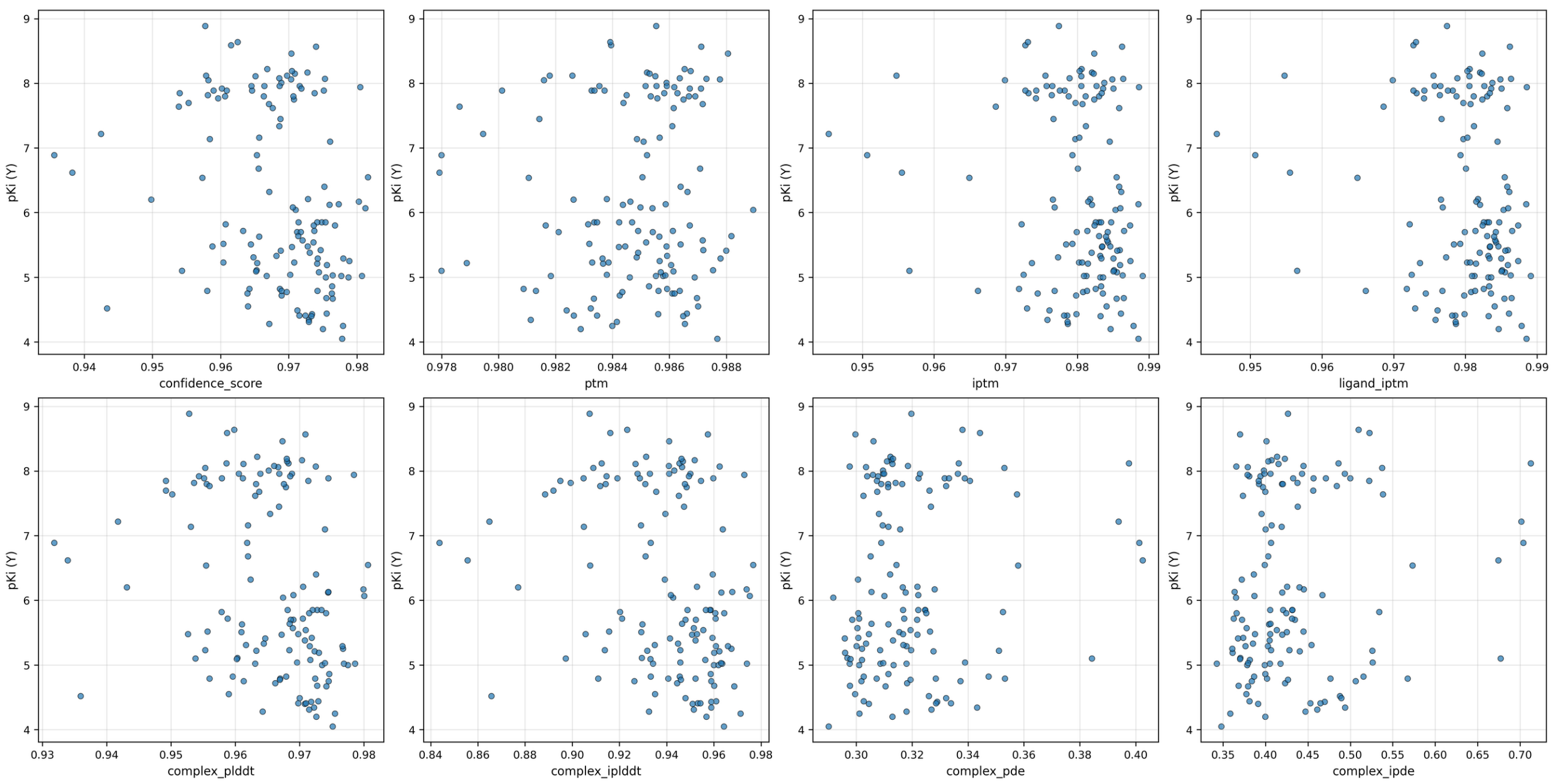

Fig. S12 Boltz-2 confidence metrics against pKi for the 132 ChEMBL retrospective inhibitors. Scatter plots of the same eight Boltz-2 confidence metrics against pK_i for the 132 known AKR1B10 inhibitors curated from ChEMBL. $|r|$ remains below 0.30 throughout.

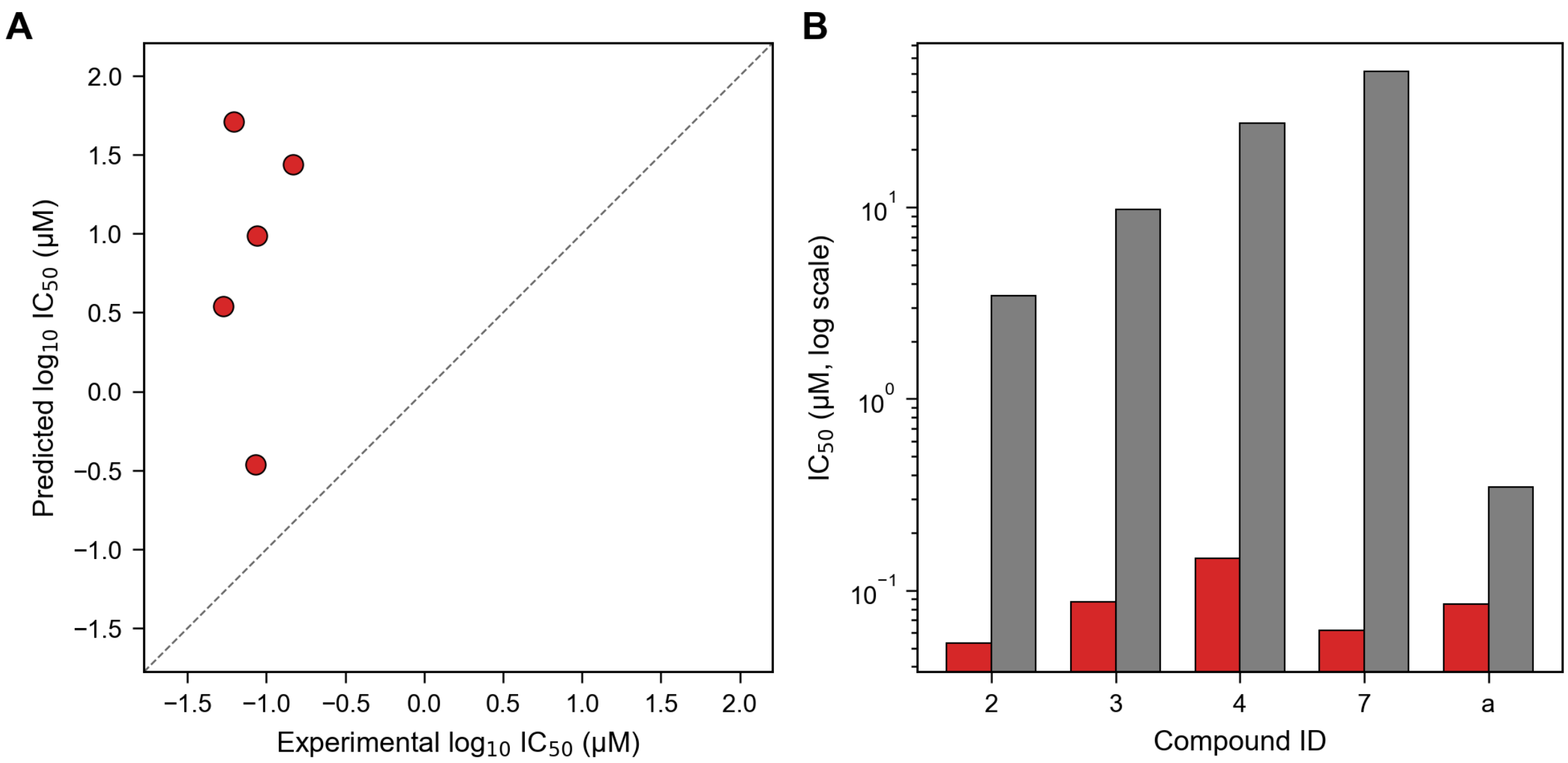

Fig. S13 Boltz-2 prediction against experiment for the five leakage-free Sun *et al* (57). AKR1B10 actives. Scatter plot of the Boltz-2 predicted $\log_{10}$ IC_{50} against the experimentally reported IC_{50} (53–147 nM) for the five natural-product compounds from *Ajuga nipponensis* reported in Sun *et al*. (57), which were independently verified across ChEMBL, PubChem BioAssay, and BindingDB to have no pre-cutoff AKR1B10 activity record. Four of the five compounds are under-predicted by two to three log units (Pearson r = 0.15, MAE = 1.93 $\log_{10}$), in contrast to the training-base ChEMBL 132 set (r = 0.54, MAE = 0.97 $\log_{10}$).

```
version: 1
sequences:
  - protein:
      id: [akr1b]
      sequence:
MATFVELSTKAKMPIVGLGTWKSPLGKVKEAVKVAIDAGYRHIDCAYVYQNEHEVGEAIQEKIQEKAVKREDLFIVSKLWPTFFERPLVRKAFEKTLK
DLKLSYLDVYLIHWPQGFKSGDDLFPKDDKGNAIGGKATFLDAWEAMEELVDEGLVKALGVSNFSHFQIEKLLNKPGLKYKPVTNQVECHPYLTQEKL
IQYCHSKGITVTAYSPLGSPDRPWAKPEDPSLLEDPKIKEIAAKHKKTAAQVLIRFHIQRNVIVIPKSVTPARIVENIQVFDFKLSDEEMATILSFNR
NWRACNVLQSSHLEDYPFNAEY
      msa: [/local_msafile_adress]
  - ligand:
      id: NADPH
      ccd: NAP
  - ligand:
      id: INH
      smiles: [*smiles]
constraints:
  - pocket:
      binder: NADPH
      contacts:
        - [akr1b, 19]
        - [akr1b, 20]
        - [akr1b, 21]
        - [akr1b, 22]
        - [akr1b, 44]
        - [akr1b, 49]
        - [akr1b, 78]
        - [akr1b, 111]
        - [akr1b, 112]
        - [akr1b, 160]
        - [akr1b, 161]
        - [akr1b, 184]
        - [akr1b, 210]
        - [akr1b, 211]
        - [akr1b, 212]
        - [akr1b, 213]
        - [akr1b, 214]
        - [akr1b, 215]
        - [akr1b, 216]
        - [akr1b, 217]
        - [akr1b, 229]
        - [akr1b, 244]
        - [akr1b, 246]
        - [akr1b, 261]
        - [akr1b, 262]
        - [akr1b, 263]
        - [akr1b, 264]
        - [akr1b, 265]
        - [akr1b, 266]
        - [akr1b, 269]
        - [akr1b, 272]
        - [akr1b, 273]
        - [akr1b, 299]
      max_distance: 4.5
  - pocket:
      binder: INH
      contacts:
        - [akr1b, 49]
        - [akr1b, 111]
```


Fig. S14 Example Boltz-2 input file in YAML format. The ligand pocket constraint (INH pocket) lists the contact residues Tyr49, His111, and Trp112 with max_distance = 4.5 Å; the cofactor pocket lists the residues within 4.5 Å of the co-crystallized $NADP^+$ (CCD code NAP). Per the Boltz-2 specification, the pocket constraint enforces an inter-atomic proximity restraint only (a maximum distance of 4.5 Å between any ligand atom and any atom of listed residue), and does not constrain side-chain rotameric states or any other conformational degrees of freedom.

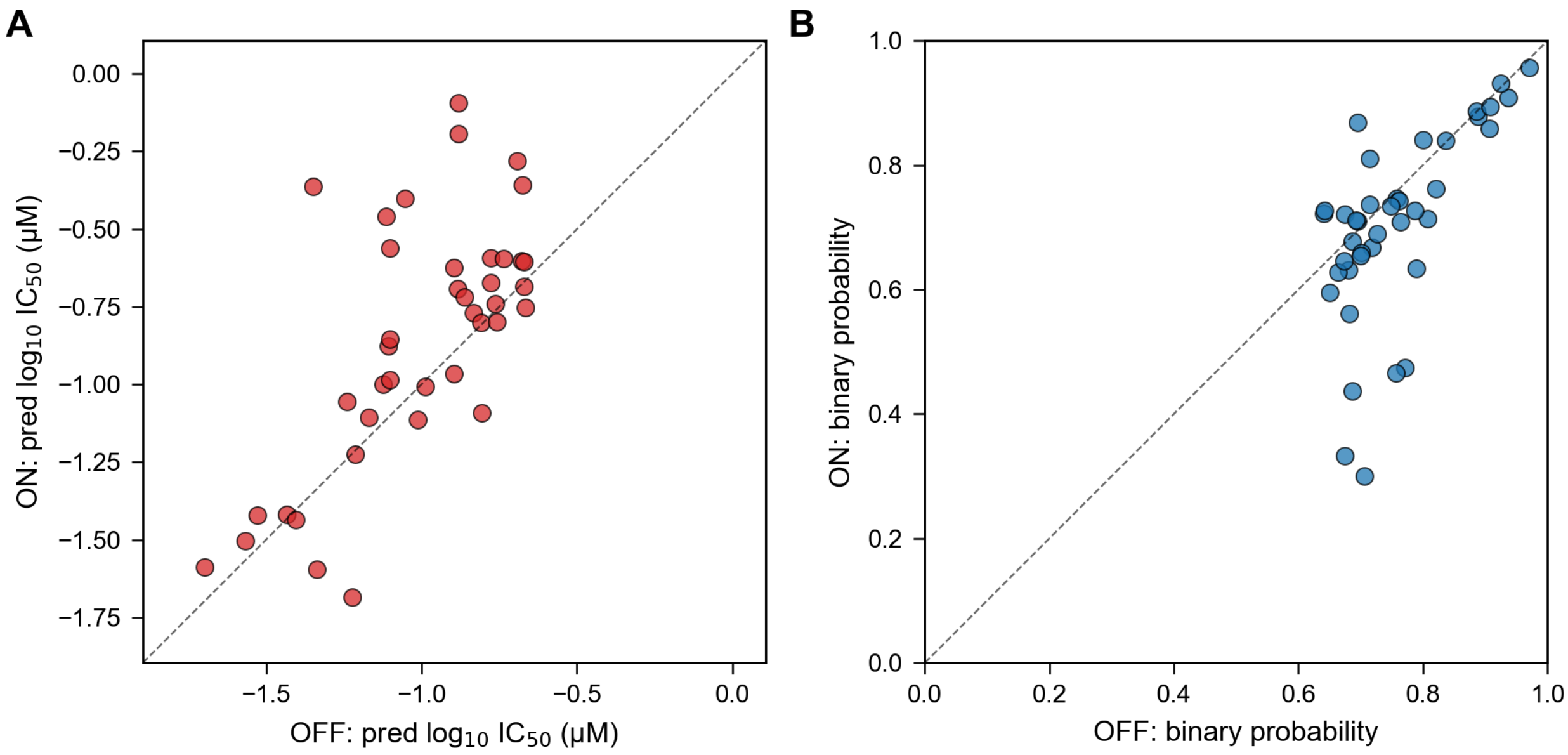

Fig. S15 Boltz-2 affinity prediction for the 40 AICE compounds under use_potentials = ON versus OFF. Scatter plots of affinity_pred_value (A) and affinity_probability_binary (B) obtained with the potential-based energy term enabled (ON, x-axis) versus disabled (OFF, y-axis). The two settings are well correlated (Pearson r = 0.69 for affinity_pred_value), with an ON-versus-OFF offset of +0.15 $\log_{10}$ in the predicted IC_{50}.

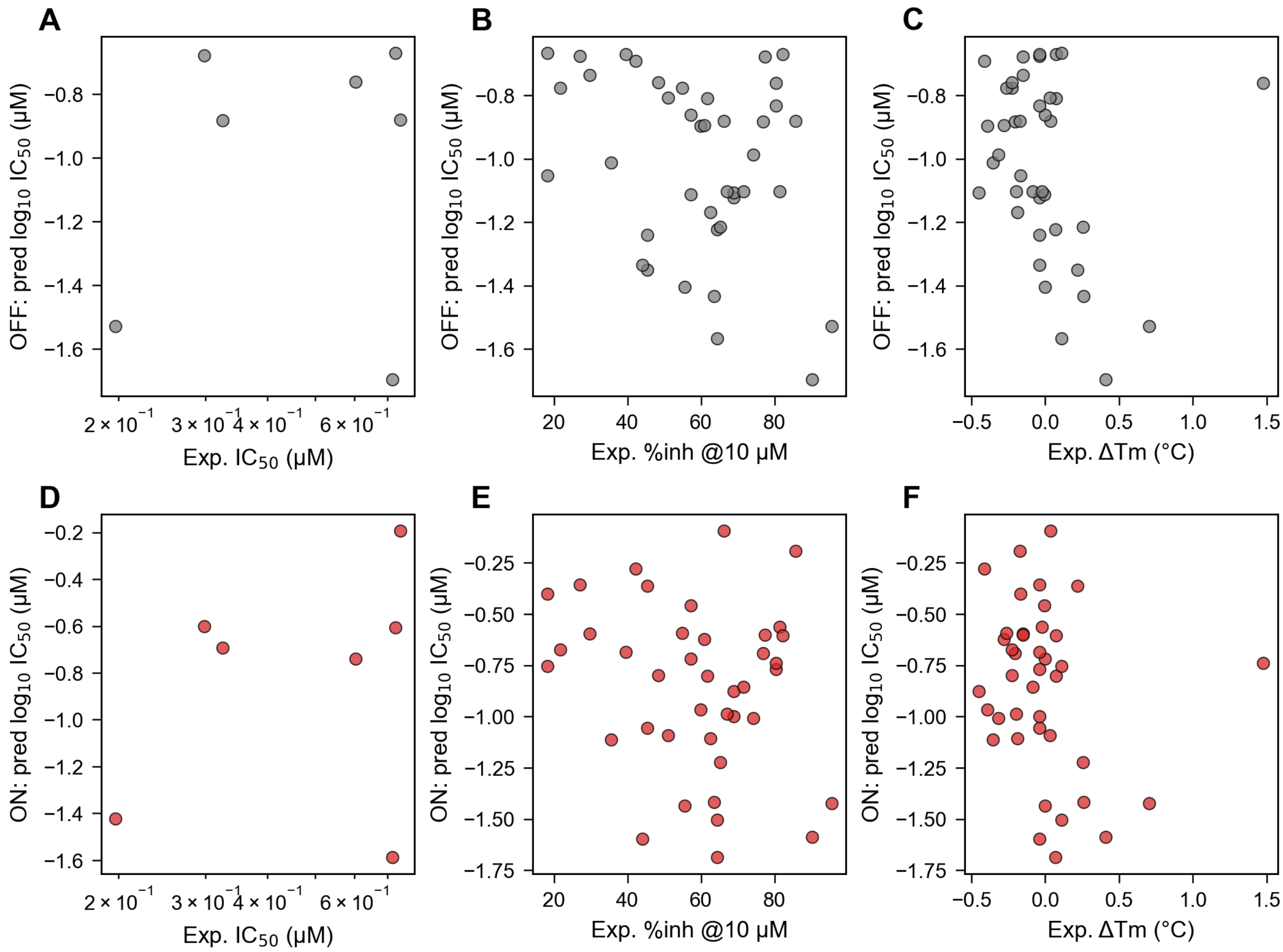

Fig. S16 Boltz-2 predicted $\log_{10}$ IC_{50} under use_potentials = ON versus OFF compared with experimental endpoints for the 40 AICE compounds. Scatter plots of the predicted $\log_{10}$ IC_{50} against (A to F) experimental IC_{50}, % inhibition at 10 µM, and TSA ΔT_m. The direction of the correlation is preserved between the two settings.

Table S1. Known inhibitors in the Boltz-2 training data. The fractions of compounds among the 132 known ChEMBL inhibitors included in the Boltz-2 training set are shown.

Database	Compounds present	Fraction
ChEMBL (document_year ≤ 2023)	132 / 132	100%
BindingDB (InChIKey connectivity)	130 / 132	98.5%
PubChem BioAssay	114 / 132	86.4%

Table S2. Cluster size of the 40 tested compounds. The cluster ID and size of each AICE compound are shown.

Compound	Cluster	cluster_size
AICE001	50	16
AICE002	6	285
AICE003	14	28
AICE004	7	6
AICE005	40	15
AICE006	0	153
AICE007	12	26
AICE008	32	6
AICE009	19	2
AICE010	27	10
AICE011	15	4
AICE012	17	7
AICE013	37	24
AICE014	1	25
AICE015	8	15
AICE016	10	12
AICE017	29	5
AICE018	5	6
AICE019	2	55
AICE020	13	10
AICE021	48	2
AICE022	44	6
AICE023	26	3
AICE024	9	4
AICE025	36	2
AICE026	4	22
AICE027	45	3
AICE028	41	2
AICE029	25	3
AICE030	16	7
AICE031	42	2
AICE032	23	5
AICE033	31	15
AICE034	3	6
AICE035	30	6
AICE036	43	8
AICE037	21	4
AICE038	20	5
AICE039	39	4
AICE040	38	5

Table S3. Threshold sensitivity sweep. Number of compounds passing affinity_probability_binary ≥ *t* for each set: the 132 ChEMBL inhibitors, the five leakage-free Sun 2024 actives, and the 40 AICE compounds. At the selected threshold *t* = 0.64, only 1 of 5 post-cutoff leakage-free Sun 2024 actives passes.

t	ChEMBL 132	Sun 2024 actives (n = 5)	AICE 40
0.50	119	2	40
0.55	111	1	40
0.60	98	1	40
0.64	91	1	40
0.68	78	1	33
0.72	60	0	20
0.76	41	0	16
0.80	29	0	11

Table S4. Physical feasibility of the predicted poses. The physical feasibility of the resulting poses of the AICE compounds and the 132 known inhibitors is investigated.

Set	n	Feasible (no clash/overlap)	Inter-atomic clash (< 2.0 Å)	Intra-ligand overlap (< 0.9 Å)
AICE prospective	40	39 / 40 (97.5%)	0 / 40	1 (AICE005)
ChEMBL retrospective	132	129 / 132 (97.7%)	3 / 132	0

Table S5. 40 test compounds. The AICE numbers and the SMILES of the 40 compounds are shown.

Compound	smiles
AICE001	CC(NC=1C=CC2=NN=C(C=3C=CSC3)N2N1)C4=CC=CC(O)=C4
AICE002	O=C1C=2C=CC(=CC2C(=O)N1C3CCC(=O)NC3=O)C4=CN(CC=5C=CC=6N=CC=CC6C5)N=N4
AICE003	CC(C)OC=1C=CC(=CC1NC(=O)COC(=O)C2=CC=CC(O)=C2)S(=O)(=O)N3CCCCC3
AICE004	CC(=O)NC=1C=CC(=CC1)C=2C=CC=C(C2)C(C)N(C)C(=O)C3=CC(=CC=C3O)S(N)(=O)=O
AICE005	CCN1N=CC(C(=O)NC2CCCC=3NC(=O)C=CC32)=C1C=4C=CC=CC4
AICE006	O=C(NC=1C=CC(F)=CC1)NC2CCN(C2)C(=O)C3=CC=C4C(=O)OC(CC4=C3)C=5C=CC=CC5
AICE007	O=S(=O)(NC1CC(CN2C=CC=N2)C1)C=3C=NC=4C=CSC4C3
AICE008	CC(C=1C=CC=C(Cl)C1F)N2C=C(N=N2)C3(O)CCOCC3
AICE009	NC=1N=CN=C2C1C(=CN2[C@@H]3O[C@H](CO)[C@@H](O)C3(F)F)C=4C=CSC4
AICE010	COC(=O)C1=CC(=NN1C)NC(=O)C(=O)NC2CCCCCC2C=3C=CC(Cl)=CC3
AICE011	N#CC1CCNCC1NC(=O)C=2C=CC=3C=NC=C(Br)C3C2
AICE012	CC=1C=CC=C(C1)CC(CO)CNC(=O)NC=2C=CC(=CC2)C3=NOC(=N3)C(F)(F)F
AICE013	N#CC=1C=C(C=CC1F)C2=CN(CC3CC4(C3)OCCO4)N=N2
AICE014	C#CCN(CCO)C(=O)C1=CC=2SC(=NC2S1)NC=3C=CC=CC3
AICE015	CC(NC(=O)NCC=1C=CN=C(C1)N(C)C)C=2C=CC(=CC2)C3=CC=CN=C3
AICE016	CN1C=NN=C1C2CCN(CC2)C=3N=NC(=CC3C(F)(F)F)C=4C=CC=CC4
AICE017	CC(O)CN(C(=O)NC1=NC=C(CC=2C=CC(Cl)=CC2F)S1)C(C)C
AICE018	CCC1=NN=C(O1)[C@H]2C[C@@H](O)CN2C(=O)C=3NC4=CC=C(Cl)C=C4C3Cl
AICE019	CC1=C(C=NN1C)S(=O)(=O)NC(=O)C2=CC=3CCCCC3S2
AICE020	OC=1C=CC=2CCC(NC=3C=CN4N=CC(Br)=C4N3)C2C1
AICE021	CCC(CO)N(C)C(=O)NC=1C=CC(=CC1)N2N=CC=3C=CC=CC32
AICE022	CC=1C=CC=2OC(=CC2C1)CN(CC3=CC=CO3)C(=O)C4=CN=CC(F)=C4O
AICE023	OCCC=1C=NC2=C(C=NN2C1)C=3C=CC=C(C3)C(F)C(F)(F)F
AICE024	COC=1C=CC=2CC(CCCC2C1)NC=3C=CN=C(C#N)N3
AICE025	CS(=O)(=O)C=1C=CC(=CC1)OC=2C=CC(=CC2)N3C(=O)NC(CC=4C=CN=CC4)C3=O
AICE026	O=C(NC=1C=CC(=CC1)C=2C=CC=CC2)[C@H]3COC(=O)N3
AICE027	OC=1C=C(F)C=2CN(CCC2C1)C=3N=CN=C4C3N=CN4CC5CCCC5O
AICE028	CN1C=NN=C1CN2CC=C(CC2)C=3C=CC=C(Cl)C3F
AICE029	C=1C=CC(=CC1)C2=NN=C(O2)C3=NN=C4CCCCCCCN34
AICE030	CCC=1C=C(N=C(N1)C=2C=CN=CC2)N3CC4(C3)CN(C)C(=O)C4(F)F
AICE031	C=CCN1C(=O)NC2C3=CC=C(C=C3OC41CCOCC24)OC
AICE032	CN1C(=O)NC(=O)C2=C1N=C(N2CC=3C=C(ON3)C=4C=CC=5OCCOC5C4)[N+]([O-])=O
AICE033	O=C1CC(CO)CN1C=2C=CC(=CC2)C=3C=CC=C4NC=CC34
AICE034	COC=1C=C(C=CC1C2=CN3C(=C2)C(=O)NCC3C4CC4)OC(F)(F)F
AICE035	CC=1C=CC(=C(F)C1)C(C)NCC(CO)CC=2C=CC=CC2C
AICE036	CN(CCOCC1CC1)C(=O)C2=CC(=NN2)C=3C=CC(Br)=CC3
AICE037	O=C1NC=2CCNCC2C=C1C3=NOC(=N3)C4CCN(CC4)S(=O)(=O)C=5C=CC=CC5F
AICE038	FC1=CN=CC(F)=C1NCCC=2C=CC=C3N=CC=CC23
AICE039	CC=1C=CC(=CC1)CC(CO)CNC(=O)C=2C=CC3=NN=C(C4CC4)N3C2
AICE040	CN(C)C(=O)C(OC(=O)C=1C=CN=C(F)C1)C=2C=CC=CC2
